# Evaluation of Two Methods for Quantification of Glycosaminoglycan Biomarkers in Newborn Dried Blood Spots from Patients with Severe and Attenuated Mucopolysaccharidosis Type II

**DOI:** 10.3390/ijns8010009

**Published:** 2022-01-21

**Authors:** Zackary M. Herbst, Leslie Urdaneta, Terri Klein, Barbara K. Burton, Khaja Basheeruddin, Hsuan-Chieh Liao, Maria Fuller, Michael H. Gelb

**Affiliations:** 1 Department of Chemistry, University of Washington, Seattle, WA 98195, USA; zherbst@uw.edu; 2 Department of Medicine, University of Washington, Seattle, WA 98195, USA; 3 National MPS Society, P.O. Box 14686, Durham, NC 27707-4686, USA; leslie@mpssociety.org (L.U.); terri@mpssociety.org (T.K.); 4 Genetics, Birth Defects & Metabolism at Ann & Robert H. Lurie Children’s Hospital of Chicago, Chicago, IL 60611, USA; bburton@luriechildrens.org; 5 Department of Pediatrics, Feinberg School of Medicine, Northwestern University, Chicago, IL 60611, USA; 6 Newborn Screening Laboratory, Illinois Department of Public Health, Chicago, IL 60604, USA; khaja.basheeruddin@illinois.gov; 7 Department of Laboratory Medicine and Pathology, University of Washington, Seattle, WA 98195, USA; liaohc6@uw.edu; 8 Genetics and Molecular Pathology, SA Pathology at Women’s and Children’s Hospital, North Adelaide 5006, Australia; maria.fuller@adelaide.edu.au; 9 School of Medicine, University of Adelaide, Adelaide 5005, Australia; 10 Department of Biochemistry, University of Washington, Seattle, WA 98195, USA

**Keywords:** newborn screening, glycosaminoglycans, mucopolysaccharidosis, Hunter syndrome, mass spectrometry, biochemical genetics

## Abstract

All newborn screening (NBS) for mucopolysaccharidosis-I and -II (MPS-I and MPS-II) is carried out via the measurement of α-iduronidase (IDUA) and iduronate-2-sulfatase (IDS) enzymatic activity, respectively, in dried blood spots (DBS). The majority of low enzyme results are due to pseudodeficiencies, and data from recent MPS-II population screenings and studies from the Mayo Clinic show that the false positive rate can be dramatically reduced by the inclusion of a second-tier analysis of glycosaminoglycans (GAGs) in DBS as part of NBS. In the present study, which focused on MPS-II, we obtained newborn DBS from 17 patients with severe MPS-II, 1 with attenuated MPS-II, and 6 patients with various IDS pseudodeficiencies. These samples were submitted to two different GAG mass spectrometry analyses in a comparative study: (1) internal disaccharide biomarkers and (2) endogenous biomarkers. For both of these methods, the biomarker levels in six patients with pseudodeficiencies were below the range measured in MPS-II patients. One patient with attenuated MPS-II was not distinguishable from severe disease patients, but all MPS-II patients were distinguishable from the reference range using both methods. The minimal differential factor (lowest GAG marker level in MPS-II samples divided by highest level in the reference range of 60 random newborns) was 3.01-fold for the internal disaccharide method. The endogenous biomarker method demonstrated an improved minimum differential of 5.41-fold. The minimum differential factors between MPS-II patients and patients with pseudodeficiencies for the internal disaccharide and endogenous biomarker methods were 3.77-fold and 2.06-fold, respectively. This study supports use of the second-tier GAG analysis of newborn DBS, especially the endogenous disaccharide method, as part of NBS to reduce the false positive rate.

## 1. Introduction

Mucopolysaccharidosis type II (MPS-II; OMIM Entry #309900), or Hunter syndrome, is a lysosomal storage disease (LSD) resulting from mutations in the iduronate-2-sulfatase gene (IDS, EC 3.1.6.13). Currently, Newborn screening (NBS) programs in Taiwan, Illinois, and Missouri are conducting population screening for MPS-II using IDS enzymatic activity in DBS as a first-tier method [1,2]. The results from these studies indicate that the prevalence of MPS-II is between 0.9 and 1.96 per 100,000 live male births [1,2,3]. Some female cases of MPS-II have been reported, though the X-linked recessive disorder is expected to mainly be found in males [4,5,6,7,8,9,10].

The measurement of enzyme activity is considered the gold-standard method for first-tier screening due to ease of implementation and flexibility for the quantification and qualification of MPS subtypes [11]. Enzyme activity measurement may be conducted either using tandem mass spectrometry (MS/MS) or a fluorescence assay of IDS activity. With both MS/MS and fluorometric activity assays, the majority of newborns with activities below the cutoffs are false positives [2,12]. In many but not all cases, false positives result from DNA variants that reduce the activity of the enzyme but not to the point that it is pathogenic (pseudodeficiencies).

The implementation of second-tier testing, such as quantification of biomarkers, has been raised as a solution to the high false positive rates associated with low enzyme activity due to pseudodeficiencies. 

Many NBS laboratories carry out DNA sequencing of the relevant gene when DBS enzyme activity is low, but the poor understanding of genotype/phenotype relationships has shown a significant number of inconclusive results. For NBS of Krabbe disease and MPS-I, the biochemical analysis of biomarkers combined with post-analysis computational tools has led to high-precision NBS with almost no false positives [13,14]. In the case of MPS-I, the measurement of short fragments of glycosaminoglycans (GAGs) leads to the elimination of most of the false positives [15,16]. Two GAG species, heparan sulfate and dermatan sulfate, accumulate when IDUA, the enzyme relevant to MPS-I, is deficient. In MPS-II, heparan sulfate and dermatan sulfate accumulate due to the lack of IDS, which removes the sulfate from the 2-position of iduronic acid residues that are a part of the GAG polymers.

Multiple methods for GAG biomarker analysis have been reported. The most commonly used method is to degrade GAG polymers in vitro with bacterial enzymes (heparinases and chondroitinases) and then measure the level of disaccharide biomarkers using liquid chromatography mass spectrometry (LC-MS/MS) [17]. We refer to this method as the “internal disaccharide method.” In the “SensiPro” method, the biomarker released from the non-reducing end of the GAG polymer after degradation with bacterial enzymes is analyzed using LC-MS/MS [18]. A second non-reducing end method was reported by Fuller and colleagues and does not require digestion with bacterial enzymes, as it measures small, non-reducing end GAG fragments that are found endogenously in patient samples [19]. We refer to this method as the “endogenous biomarker method”.

In our recent study, we obtained newborn DBS from patients already diagnosed with MPS-I and systematically studied all three GAG quantification methods described above [16]. We found that the endogenous biomarker method gave by far the largest differentiation between MPS-I patients and healthy newborns.

In the current study, we explore the internal disaccharide and endogenous biomarker methods for the measurement of accumulated GAGs by LC-MS/MS in newborn DBS from diagnosed MPS-II patients and from MPS-II pseudodeficiencies. We did not study the SensiPro method because sample preparation is considerably more complex than with other methods, and it did not outperform the endogenous biomarker method when analyzed on MPS-I samples [16].

## 2. Materials and Methods

Studies with DBS were approved by the Univ. of Washington IRB (number 5580, approved 7 January 2020). Staff at the National MPS Society used their registry to reach out to families with MPS-II patients for consent to participate in the study. In appropriate states, family members used publicly available protocols to request a stored newborn DBS from their state NBS laboratory. De-identified information about the affected patient and the DBS was sent to the Gelb laboratory. Clinical information and genotype (when available) for all patients are provided in Tables S1–S3. The state-specific DBS storage conditions are also provided. 

We also worked with the Illinois NBS laboratory to obtain de-identified newborn DBS that had below-cutoff IDS activity but were later shown to present MPS-II pseudodeficiencies based on genotype and a lack of well-elevated GAGs in urine, measured during follow-up diagnostic testing. As far as we know, these individuals remain MPS-II asymptomatic. Information about these patients is provided in Table S2.

Detailed methodologies for GAG analyses are provided as Supplementary Material.

## 3. Results

### 3.1. GAG-Derived Biomarkers and Methods of Detection

The IDS enzyme is responsible for the cleavage of GAG oligomers with a 2-O-sulfated α-L-iduronic acid non-reducing end (NRE), a crucial step in the breakdown of the GAG species heparan sulfate and dermatan sulfate (also known as chondroitin sulfate B). As a result, deficiencies in IDS enzyme activity associated with MPS-II phenotypes leads to an accumulation of heparan sulfate and dermatan sulfate in the lysosomes of cells throughout the body. The measurement of these GAGs has been explored using multiple methods, and current NBS programs report GAG biomarker levels as part of genetic diagnostic assessments following first-tier IDS activity population screens [2,3]. 

**Figure 1:**
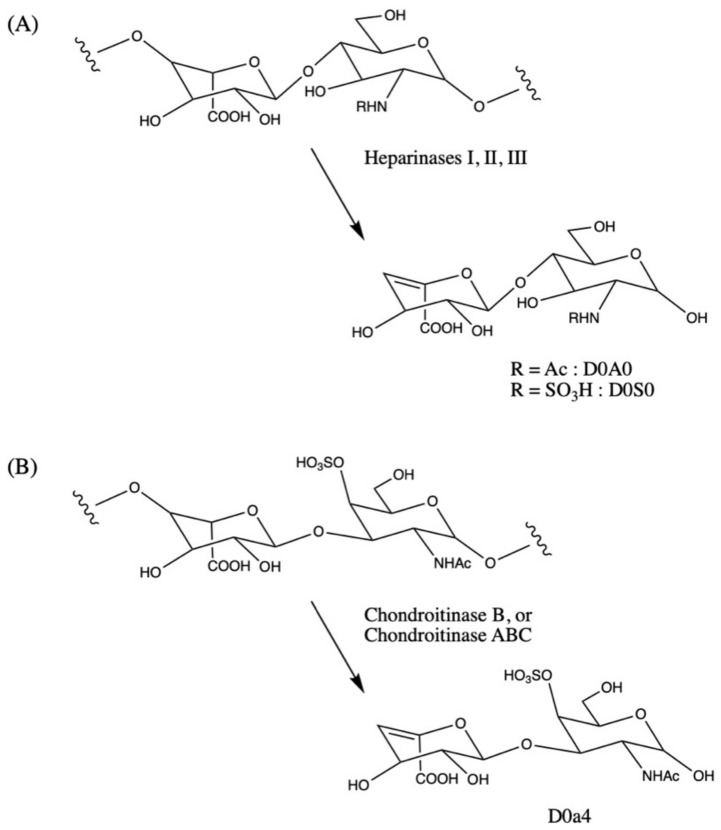
(**A**) Heparan sulfate disaccharide repeat unit showing sulfation or acetylation on the amino group. The 2-position of the iduronic acid residue can be sulfated (not shown). Degradation with bacterial lyases heparinases I, II, and III yields internal disaccharides containing unsaturated uronic acids (D0A0 and D0S0). (**B**) Dermatan sulfate (chondroitin sulfate B) disaccharide repeat unit showing iduronic acid residue and sulfation at the 4-O position of N-acetylgalactosamine (GalNAc), a major disaccharide in dermatan sulfate. Digestion with the bacterial lyases chondroitinase B or chondroitinase ABC yields the unsaturated internal disaccharide, D0a4. These internal disaccharides are detected using LC-MS/MS without further derivatization.

In the internal disaccharide method, samples are treated with a mixture of three heparan sulfate lyases (heparinases I, II, and III from *Flavobacterium heparinum*), and a dermatan sulfate lyase (chondroitinase B from *Flavobacterium heparinum* or chondroitinase ABC from *Proteus vulgaris*). Each lyase has high specificity for its GAG substrates. The use of three bacterial heparinases (I, II, and III) is commonly reported, and presumably leads to a more complete degradation of heparan sulfate. Each lyase breaks the relevant GAG oligomers into multiple species of disaccharide fragments containing unsaturated uronic acids (ΔUA), which are detectable using LC-MS/MS without derivatization. Not all fragments are identical due to various degrees of sulfation throughout the GAG polymer. Three disaccharides of interest are targeted, named by previously published conventions [20], including D0A0 and D0S0 from heparan sulfate and D0a4 from dermatan sulfate (see Figure 1). Additional naming conventions for these internal disaccharide biomarkers are used across laboratories, so a reference table is given in the Supplementary Materials (Table S4).

**Figure 2:**
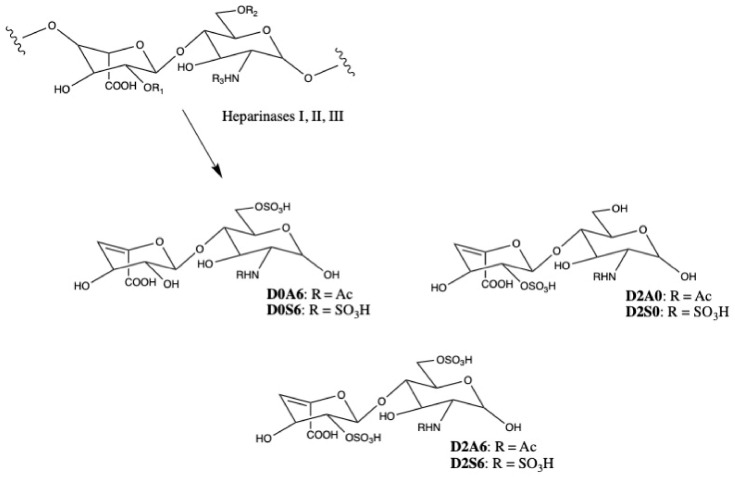
Six internal disaccharide heparan sulfate biomarkers were investigated, in addition to D0A0 and D0S0. Heparan sulfate disaccharide repeat unit showing sulfation or acetylation on the amino group, potential 6-O-sulfation of the glucosamine residue, and potential 2-O-sulfation of the iduronic acid residue. Degradation with bacterial lyases heparinases I, II, and III yields internal disaccharides containing unsaturated uronic acids.

The three disaccharides shown in Figure 1 represent a small selection of known internal disaccharides from heparan sulfate, which were detected via LC-MSMS after aniline labeling by Lawrence et al. in 2008 [21]. In 2014, Tomatsu et al. evaluated unlabeled D0A0, D0A6, and D0S0 from heparan sulfate and unlabeled D0a4 from dermatan sulfate by the internal disaccharide method [17]. In addition to D0A0 and D0S0, we investigated six additional heparan sulfate internal disaccharides to determine whether they should be quantified in newborn DBS for MPS-II biomarker screening. Using commercial unsaturated uronic acid standards from Iduron (Manchester, UK), the multiple-reaction monitoring mass spectrometry parameters were determined in negative mode ESI for D0A6 (458 > 175), D0S6 (496 > 395), D2A0 (458 > 237), D2S0 (496 > 235), D2A6 (538 > 458), and D2S6 (577 > 458) (see Figure 2). Of these six, D2A6 and D2S6 showed poor ionization, likely due their high sulfation resulting in premature fragmentation in the ESI source. The n-sulfated disaccharides showed 2–10x lower peak areas compared to their n-acetylated analogues (e.g., D0S0 vs. D0A0, D0S6 vs. D0A6, and D2S0 vs. D2A6). Ionization parameters for each internal disaccharide standard were optimized using a Waters Xevo TQ-S, and multiple-reaction monitoring parameters were selected based on MSMS fragments observed in product scans of each. All LC-MSMS methods and conditions are given as Supplementary Materials.

Urine and DBS from MPS-II-diagnosed newborns and healthy newborns were digested with bacterial heparinases according to the internal disaccharide method (see the Supplementary Materials), and levels of the six disaccharides were measured via LC-MSMS. Although a peak was identified for each disaccharide in the commercially available mixture of heparan-sulfate-derived disaccharides, D0S6, D2S0, D2A6, and D2S6 were not detected in healthy or MPS-II newborn urine or DBS. The D0A6 and D2A0 disaccharides were present and slightly elevated in newborn MPS-II-diagnosed urine (*n* = 1), but elevation was not seen in MPS-II DBS, and often, the peaks were absent (data not shown). As a result, we are confident that D0A0 and D0S0 are the best internal disaccharide biomarkers for heparan sulfate in MPS-II newborn DBS. Alternative internal disaccharide biomarkers for dermatan sulfate were not investigated in this study.

A second method quantifies accumulated endogenous GAG-derived biomarkers, which have been previously identified in urine from MPS patients, including MPS-II [19]. These endogenous biomarkers result from the degradation of accumulated GAGs by endogenous enzymes in humans. The origin of endogenous biomarkers is explained by two reported pathways for endogenous GAG degradation: one pathway is the sequential digestion of GAGs at the non-reducing end by endogenous exohydrolases, and one pathway breaks linkages between internal GAG residues by unidentified endoenzymes [22]. In MPS-II, for example, IDS enzyme deficiency halts sequential GAG digestion at the iduronic acid 2-sulfate NRE, then an unidentified enzyme cleaves the GAG at an internal linkage. This likely results in two products: a short-chain biomarker containing the NRE, and the remainder of the GAG polysaccharide, which continues sequential digestion until the process above repeats. Presumably, this process results in a large number of short-chain GAG biomarkers in the body with identical NRE residues. We expect a different NRE to accumulate in each type of MPS patient. This would explain why a unique endogenous biomarker is observed in the urine from each type of MPS patient [19]. 

For MPS-II, the biomarker previously reported in patient urine is UA-HNAc(1S) late retention time, which designates a disaccharide with one uronic acid residue, one N-acetyl hexosamine residue, and a single sulfation, but the exact structure is not known [19]. Given that IDS is deficient, the likely structures are iduronic acid 2-O-sulfate linked to N-acetylglucosamine via an α(1,4) linkage (heparan sulfate derived), or iduronic acid 2-O-sulfate linked to N-acetylgalactosamine via a β(1,3) linkage (dermatan sulfate derived).

Table 1 summarizes the methods for GAG analysis investigated in this study. Both methods rely on chromatographic technologies for the retention and resolution of highly polar, structurally similar analytes in reverse-phase. The biomarkers analyzed in the internal disaccharide method are resolved via a porous graphitic carbon column (Hypercarb Graphitic Column, Thermo Scientific, Waltham, MA, USA) without derivatization. In the endogenous disaccharide method, analytes are derivatized with 1-phenyl-3-methyl-5-pyrazolone (PMP). This step improves chromatographic resolution by decreasing polarity, and the PMP provides predictable fragmentation during MS/MS detection. The derivatized endogenous biomarkers are resolved via a perfluorophenyl (PFP) column (Pursuit PFP Column, Agilent, Santa Clara, CA, USA), where analyte retention is driven by interactions between stationary-phase fluorine groups and highly polar functional groups on the analytes.

Currently, chemically identical, isotopic internal standards are not available for either method described in this report. Instead, structural analogues are used as internal standards for the quantification of biomarkers in both methods (Table 1). This approach comes with some disadvantages. Without LC coelution of the internal standard and the analyte, the effect of different matrix components on analyte ionization cannot be accounted for. Secondly, response factor ratios for each analyte and the internal standard are not assumed to be unity. Finally, run-to-run variations in the mass spectrometer’s condition can impact the response factors of the analytes and internal standard. 

For the internal disaccharide method, we use a mass spectrometry standard (MS standard) to reduce these disadvantages. This standard is prepared in one lot, then is aliquoted and stored for long-term use. The MS standard contains equal moles of all internal disaccharides (available commercially) and the internal standard. Moles of each internal disaccharide and the internal standard are determined using quantitative proton nuclear magnetic resonance (qNMR) rather than using gravimetric analysis, which can be problematic for absolute quantification [23]. The injection of this standard in every LC-MS/MS run provides a response factor ratio, which is the measured signal per mole of analyte, divided by the measured signal per mole of internal standard. This response factor ratio can be applied as a factor to correct for the difference in analyte/internal standard response and to correct for inter-assay variations. See the Supplementary Materials for complete information about the preparation and use of the mass spectrometry standard.

### 3.2. Results Using the Internal Disaccharide Method

The internal disaccharide method has been published in multiple papers by Tomatsu’s group, for example [24], and by other groups [15,25]. Our initial investigations of the method were based on a protocol reported by Kubaski et al. in 2017 [26]. The original method dictates the overnight incubation of DBS samples in buffer with heparinases and chondroitinase B. Prior to analysis via LC-MS/MS, the analytes are isolated via ultra-filtration using a 96-well filter plate. In our previous study, we showed that the ultra-filtration step can be replaced [16]. In this new method, we quench the enzymatic digests with methanol to precipitate proteins, remove insolubles via centrifugation, then collect and dry the supernatant for reconstitution in 100% water prior to LC-MS/MS analysis. The results from a recent study by Tóth et al. further demonstrate that changes to the original method will not affect stability and recovery of the dermatan sulfate disaccharide, D0a4 [27]. Stability and recovery issues have not been investigated for heparan sulfate disaccharides D0A0 and D0S0.

**Figure 3:**
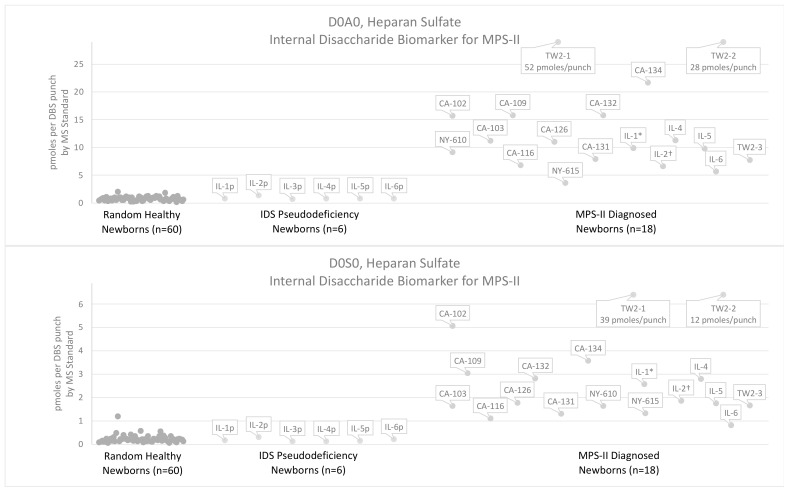
Levels of heparan-sulfate-derived (D0A0, D0S0) internal disaccharides measured in DBS from 60 random healthy newborns, 6 newborns with IDS pseudodeficiency, and 18 newborns diagnosed with MPS-II. Biomarkers measured using the internal disaccharide method with heparinase digestion in buffer. Note that one random healthy newborn DBS showed high D0S0 levels compared to the healthy newborn pool. TW2-1 and TW2-2 showed very high D0A0 and D0S0 levels above the range of the plots (52 pmoles D0A0/punch and 28 pmoles D0A0/punch, and 39 pmoles D0S0/punch and 12 pmoles D0S0/punch). The D0A0 and D0S0 levels for these two samples are labeled in the plots. * DBS from patient with undetermined phenotype, † DBS from patient with attenuated phenotype, “p” DBS from newborn that failed tier-1 IDS screening, pseudodeficiency identified, no MPS-II diagnosis, Label key and DBS storage conditions: CA = California (frozen), IL = Illinois (frozen), TW = Taiwan (frozen), NY = New York (ambient).

Figure 3 shows levels of internal disaccharides derived from heparan sulfate (D0A0 and D0S0) in newborn DBS from 18 MPS-II patients (16 severe, 1 attenuated, and 1 undetermined phenotype) and 60 random newborns. Units of pmoles biomarker per DBS punch by the MS standard were obtained using chondrosine as an internal standard and by correcting for the biomarker-to-chondrosine response factor using the previously described MS standard (see Supplemental Methods for full details). Samples labeled NY were stored at ambient temperature, and samples labeled CA, IL, and TW were frozen and stored.

**Figure 4:**
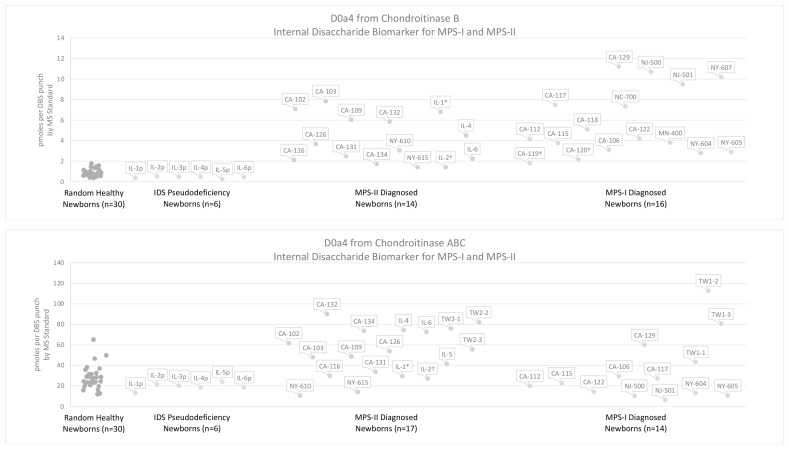
Levels of dermatan-sulfate-derived (D0a4) disaccharide measured in DBS from 30 random healthy newborns, six newborns with IDS pseudodeficiencies, newborns diagnosed with MPS-II, and newborns diagnosed with MPS-I using the internal disaccharide method with chondroitinase B or chondroitinase ABC digestion in buffer. As a result of limited samples, not all MPS-I and MPS-II newborn samples were tested with both enzymes. Results from 13 severe MPS-I, 2 attenuated MPS-I, and 30 random newborns incubated with chondroitinase B were previously published [16]. * DBS from patient with undetermined phenotype, † DBS from patient with attenuated phenotype (MPS-I or MPS-II), “p” DBS from newborn that failed tier-1 IDS screening, pseudodeficiencies identified, no MPS-II diagnosis, Label key and DBS storage conditions: CA = California (frozen), IL = Illinois (frozen), MN = Minnesota (frozen), TW = Taiwan (frozen), NJ = New Jersey (frozen), NY = New York (ambient).

In our analysis of MPS-II newborns, we evaluated two lyases which degrade dermatan sulfate: chondroitinase B and chondroitinase ABC. Figure 4 compares the levels D0a4 liberated with chondroitinase B versus those liberated with chondroitinase ABC in newborn DBS from MPS-II patients, MPS-I patients (previously published), and 60 random healthy newborns (30 previously published) [16]. Table S7 gives D0a4 levels measured in newborn MPS urine using chondroitinase B or chondroitinase ABC. Ultimately, we determined that chondroitinase B is the most selective enzyme for the liberation of D0a4 in MPS-II newborn DBS compared to 30 random controls. Only D0a4 data from chondroitinase B are considered in the discussion of results. All numerical data for DBS analyzed via the internal disaccharide method are reported in the Supplementary Materials, Table S6.

### 3.3. Results Using the Endogenous Biomarker Method

In our initial approach to identifying the MPS-II endogenous biomarkers, the endogenous disaccharide biomarker, UA-HNAc(1S) late retention time, was measured as its PMP derivative in patient urine samples from 10 MPS subtypes (MPS-I, -II, -IIIA, -IIIB, -IIIC, -IIID, -IVA, -IVB, -VI, and -VII). Consistent with the previous report [19], we found relatively high levels of this marker in newborn MPS-II urine (Table S5). Next, we studied patient fibroblasts from seven MPS subtypes (MPS-I, -II, -IIIA, -IIIB, -IVA, -VI, and -VII) and found high levels of the endogenous disaccharide in MPS-II fibroblasts (Table S5). 

In our previous publication, we identified endogenous disaccharide UA-HNAc(1S) early retention time as a biomarker that is elevated in both attenuated and severe MPS-I newborn DBS [16]. As previously described, the signature endogenous biomarker for MPS-II is an isomer of the MPS-I marker, and the MPS-I and MPS-II biomarker peaks elute from the LC column with different retention times [19]. To differentiate between them, we refer to the MPS-II biomarker as UA-HNAc(1S)-late, and the MPS-I biomarker as UA-HNAc(1S)-early. Peaks for both the UA-HNAc(1S)-early and the UA-HNAc(1S)-late biomarkers are detected using the same multiple-reaction monitoring (MRM) trace. 

In both fibroblasts and urine, UA-HNAc(1S)-late appears to be elevated in both MPS-I and MPS-II patients (Table S5). Saville et al. also reported elevation of the UA-HNAc(1S)-late marker in MPS-I urine [16]. This elevation in MPS-I samples is likely explained by tailing of the UA-HNAc(1S)-early biomarker peak in the MRM trace. In MPS-I samples, the UA-HNAc(1S)-early peak is seen to elute 0.4 min before the expected retention time of the UA-HNAc(1S)-late peak, which is seen in MPS-II samples. Additionally, the peak response for UA-HNAc(1S)-early in MPS-I samples is 10× greater than that of UA-HNAc(1S)-late in MPS-II samples. In MPS-I patient samples, tailing from the early biomarker peak contributes MS/MS signal in the region of the chromatogram used for integration of the later peak in MPS-II patient samples (see Figure S1). The 10× higher signal of the MPS-I early biomarker peak means that the tailing appears as significant ion counts in the range of the late peak’s retention time for MPS-I newborn DBS. However, in MPS-II newborn DBS, there are no counts in the region of the early marker, only in the range of the late marker. Thus, MPS-I and MPS-II newborn DBS are easily distinguished from each other.

When analyzing the urine and fibroblast data, a second elevated endogenous biomarker was noted. The trisaccharide UA-HNAc-UA(1S) was analyzed but not reported as an MPS-II marker by Fuller’s group [19]. This is likely because the peak for this trisaccharide marker appears clearly in both MPS-I and MPS-II urine and fibroblasts (Figure S1). This second MPS-II biomarker may be a useful in discriminating between samples where the MPS-II disaccharide level is close to the cutoff. In this paper, both the disaccharide and trisaccharide endogenous biomarkers were analyzed in newborn MPS-II DBS samples.

**Figure 5:**
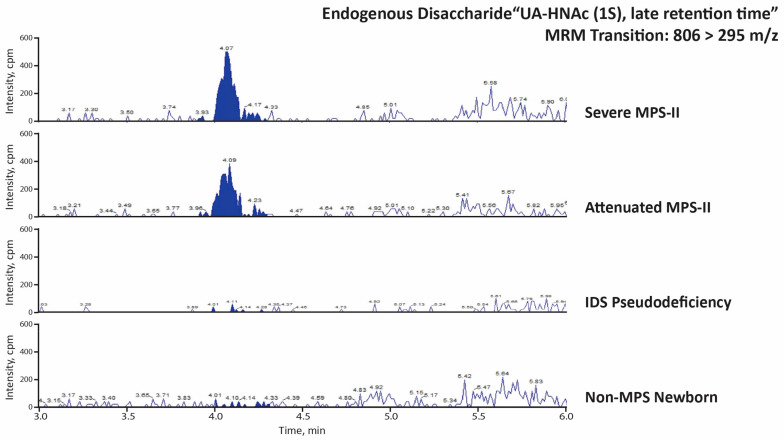
LC-MS/MS traces (MRM signal response versus time) for the PMP-derivatized endogenous disaccharide marker UA-HNAc (1S) late retention time (transition: 806 > 295 *m*/*z*), using newborn DBS from indicated patients.

**Figure 6:**
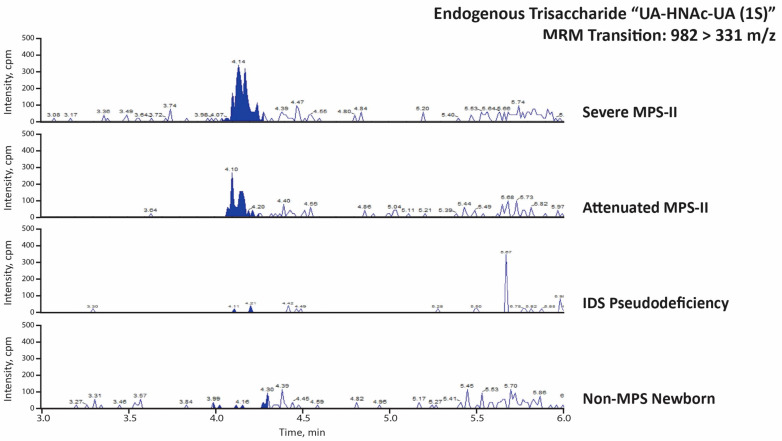
LC-MS/MS traces (MRM signal response versus time) for the PMP-derivatized endogenous trisaccharide marker UA-HNAc-UA (1S) (transition: 982 > 331 *m*/*z*) using newborn DBS from indicated patients.

The MPS-II endogenous disaccharide and trisaccharide biomarkers were analyzed in newborn DBS from 19 MPS-I patients, 16 severe MPS-II patients, 1 attenuated MPS-II patient, 1 MPS-II patient with undetermined severity, 6 patients with IDS pseudodeficiencies, and 60 random newborns (presumably non-MPS). Figure 5 and Figure 6 show typical LC-MS/MS chromatograms from each MPS-II biomarker using two 3 mm punches from a newborn patient with severe MPS-II, a newborn patient with attenuated MPS-II, a newborn patient with an IDS pseudodeficiency, and one random newborn control. The MPS-II endogenous disaccharide (UA-HNAc(1S)-late) is shown as a clear peak in the severe and attenuated MPS-II newborn DBS, while the signal for the pseudodeficiency newborn and the random newborn sample are essentially background noise (Figure 5). The MPS-II endogenous trisaccharide’s peak is half as strong as the endogenous disaccharide in the same severe and attenuated MPS-II newborn DBS. The endogenous trisaccharide signal in the IDS pseudodeficiency newborn and the random newborn control is essentially noise-level (Figure 6).

**Figure 7:**
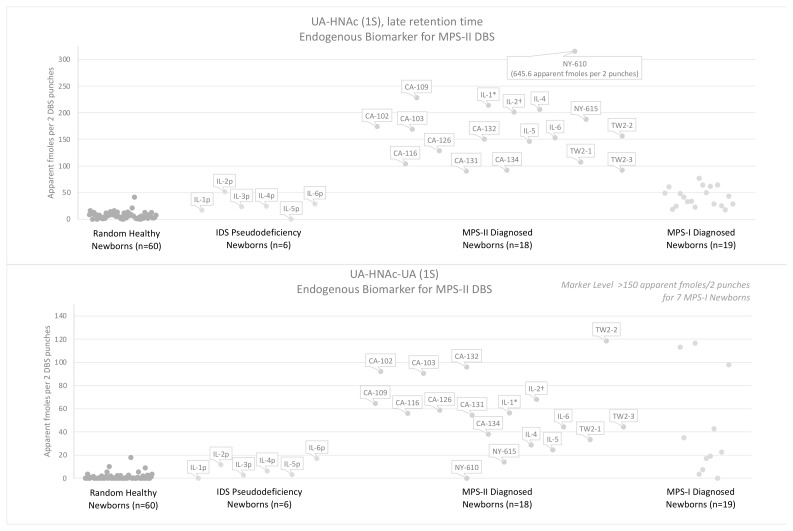
Levels of the MPS-II endogenous disaccharide UA-HNAc(1S)-late and trisaccharide UA-HNAc-UA(1S) biomarker PMP derivatives in newborn DBS from 60 random newborns (non-MPS), from 6 newborns with an IDS pseudodeficiency, from 16 newborns with severe MPS-II, 1 newborn with undetermined MPS-II phenotype, 1 newborn with attenuated MPS-II, and 19 newborns with MPS-I (previously reported [16]). * DBS from patient with undetermined phenotype, † DBS from patient with attenuated phenotype, Label key and DBS storage conditions: CA = California (frozen), IL = Illinois (frozen), TW = Taiwan (frozen), NY = New York (ambient).

Figure 7 shows the levels of each MPS-II endogenous biomarker as its PMP derivative measured in two 3 mm DBS punches. A control group of 60 random newborns (presumably non-MPS) are compared to 18 newborn MPS-II DBS (sample information is given in Table S1), 6 newborn IDS pseudodeficiency DBS (sample information given in Table S2), and 19 newborn MPS-I DBS (sample information give in Table S3). One of the eighteen MPS-II DBS is from an attenuated MPS-II newborn (IL-2), and one DBS is from a patient with an inconclusive genotype (IL-1) because the genotype is neither neuronopathic nor of a known attenuated mutation. Apparent fmoles per two 3 mm DBS punches is given based on use of PMP-derivatized chondroitin disaccharide di-4S as an internal standard. We use apparent to indicate that fmoles of a biomarker is calculated assuming a biomarker-to-internal standard relative response factor of one (and we have no way to measure the true value of this relative response factor). Samples labeled NY were stored at ambient temperature, and samples labeled CA, IL, and TW were frozen and stored. All numerical data for DBS analyzed via the endogenous biomarker method are reported in the Supplementary Materials, Table S6.

**Figure 8:**
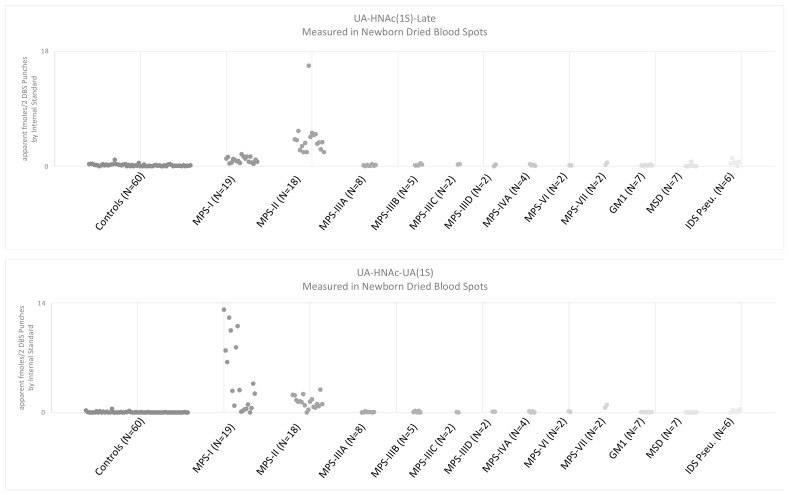
Levels of each MPS-II endogenous biomarker are compared between newborn DBS from 60 random healthy controls, 19 newborns with MPS-I, 18 newborns with MPS-II, 8 newborns with MPS-IIIA, 5 newborns with MPS-IIIB, 2 newborns with MPS-IIIC, 2 newborns with MPS-IIID, 4 newborns with MPS-IVA, 2 newborns with MPS-VII, 7 newborns with GM1 Gangliosidosis, 7 newborns with multiple sulfatase deficiency (MSD), and 6 newborns with IDS pseudodeficiency.

Figure 8 shows the levels of each MPS-II endogenous biomarker as its PMP derivative measured in two 3 mm DBS punches from various newborn DBS. These figures show that the UA-HNAc(1S)-late biomarker is elevated in MPS-II above all other controls reported. The elevation of both UA-HNAc(1S)-late and UA-HNAc-UA(1S) is characteristic of MPS-II newborns. The elevation of both UA-HNAc(1S)-early and UA-HNAc-UA(1S) may be characteristic of MPS-I newborns. The UA-HNAc-UA(1S) marker is a valuable control that may help decrease false positives when considered in addition to the UA-HNAc(1S)-late disaccharide biomarker (discussed later).

## 4. Discussion

In this study, we reported the levels of GAG-derived biomarkers from newborn MPS-II patient DBS. The main goal of this paper was to see if GAG-derived biomarkers in newborn DBS could be used as a second-tier screening in NBS to follow the measurement of IDS activity in DBS. In this study, we measured the selectivity of GAG-derived biomarker measurement in discriminating between DBS from healthy newborns, newborns with IDS pseudodeficiency, and newborns diagnosed with MPS-II. Fortunately, some NBS laboratories store residual DBS for years, and these can be retrieved with consent from the families of the affected patient. Patient registries maintained by disease foundations, in this case the National MPS Society, are critical for obtaining such samples. The current report was based on samples received during the last ~3 years, and we continue to receive newborn DBS from all types of MPS patients in support of this research. Given that NBS for MPS-II has started, and that this disease has been nominated for inclusion on the Recommended Uniform Screening Panel, we felt that it was important to disclose the MPS-II data we have obtained to date.

A second purpose of this study was to evaluate two previously reported methods for the analysis of GAG biomarkers in attenuated and severe MPS-II newborn DBS. The first method, the internal disaccharide method, is considered the “classic” approach to analyzing GAG levels in DBS. The second method, the endogenous biomarker method, is less widely used and was only recently reported for the measurement of GAG biomarkers in newborn urine alone [19]. For NBS, it was important to expand this method for the analysis of newborn DBS. This would allow the measurement of biomarkers prior to contacting families of newborns who fail first-tier IDS screening. DNA sequencing of the IDS gene starting from the DBS sample can be carried out, but reports to date show that the genotype information is often inconclusive due to the presence of variants of unknown pathogenic significance, or the inability to integrate the effect of multiple partially penetrant variants. 

The methodologies investigated in this study exclusively rely on the detection of GAG fragments using LC-MS/MS. Mass spectrometric analysis affords high sensitivity and specificity compared to methods that indiscriminately measure intact GAG polymers (e.g., analysis with fluorometric dyes). However, it should be noted that mass spectrometers with detection sensitivities higher than those typically used in NBS laboratories are needed for GAG biomarker detection using LC-MS/MS. Thus, it may be more practical for NBS laboratories to send those DBS that display low lysosomal enzymatic activity to qualified reference laboratories rather than establishing the method in house. This is what is done for all NBS laboratories now, using GAG biomarker analysis to support NBS of MPS-I.

The enzymatic or chemical fragmentation of full-length, endogenous GAG polymer chains is important for quantification. Fragmentation is important because the structural variation between full-length molecular GAG species is enormous due to highly varied sulfation and acetylation patterns across the full-length polymer. Additionally, GAG species exist at various stages of endogenous enzymatic processes, including: N-deacetylation, N-sulfation, epimerization between uronic and iduronic acid, and O-sulfation at various positions [21]. The partial endogenous digestion of these full-length chains further increases the vast number of unique molecular species that can exist simultaneously in the body. The methanolysis of GAG polymers is one in vitro method for GAG fragmentation which has been used instead of enzymatic cleavage by bacterial lyases [28]. We only studied the enzymatic method since both methods look at internal disaccharides rather than the non-reducing end and are expected to show similar discrimination between MPS patients and non-affected individuals.

After digestion with bacterial lyases, levels of the internal disaccharides D0A0 and D0S0 were above the reference range for MPS-II newborn DBS (Figure 3). Levels of D0A0 and D0S0 markers in the attenuated MPS-II patient sample fell into the lower range of severe MPS-II samples, but the sample is not distinguishable from severe patients. It is important to note that one of the 60 random newborns had a slightly elevated D0A0 level, and the D0S0 marker showed 2-fold elevation above other random newborns. This outlier was previously described in our MPS-I paper [16] and may represent a newborn with previously undetected enzyme deficiency. However, we have no additional data about this newborn.

The dermatan-sulfate-derived D0a4 was measured in MPS-I and MPS-II DBS after treatment with chondroitinase B (Figure 4). The D0a4 marker level in MPS-I and MPS-II DBS overlapped with the reference range (30 random newborns). Overlap of D0a4 levels with the reference range occurred for 6% of MPS-I samples (1/16) and 14% of MPS-II samples (2/14) treated with chondroitinase B. 

Levels of the internal disaccharide biomarkers were also measured in six IDS pseudodeficiency newborn DBS that had below-cutoff IDS activity in the IL NBS laboratory but were later reported as pseudodeficiencies based on genotype (see Table S2 for sample information). The internal disaccharides levels (D0A0, D0S0, and D0a4) were found to overlap with the reference range for these six patients (see Figure 3 and Figure 4).

We define the “minimum differential” as the fold increase in biomarker level between the lowest level in patient samples versus the highest level in random controls. The fold difference for severe MPS-II newborns was 3.02-fold for D0A0, 1.45-fold for D0S0, and 1.10-fold for D0a4. For the single attenuated MPS-II newborn, the fold differences were 3.51-fold for D0A0 and 3.31-fold for D0S0, while D0a4 was <1.00-fold (i.e., overlap with the reference range). Minimum differentials were also calculated between MPS-II newborn DBS and the six IDS pseudodeficiency newborn DBS. The minimum differential between the pseudodeficiency newborns and MPS-II newborns was 3.98-fold for D0A0, 2.75-fold for D0S0, and 3.34-fold for D0a4. Due to uncertainties about GAG stability, two MPS-II newborn DBS stored at ambient conditions for 7.8 years and 14 years were excluded from the minimum differential calculations (NY-610 and NY-615, respectively). The random newborn outlier described above also was excluded from these calculations of minimum differential.

The fold increases reported above are similar to GAG biomarker levels in MPS-II newborn DBS reported in the literature. An MPS-II-diagnosed newborn DBS with neuronopathic phenotype was compared to 59 newborn controls by de Ruijter et al. in 2012. The minimum differentials for the single patient were 3.38-fold for D0A0 and 2-fold for D0a4 (D0S0 levels were not analyzed) [25]. In their 2017 pilot study, Tomatsu and colleagues reported data for two MPS-II newborn DBS using cutoff values determined with 2862 controls. Minimum differentials between samples and the established cutoff values were 2.67-fold for D0A0, 1.48-fold for D0S0, and 1.03-fold for D0a4 [29]. Data with non-newborn MPS-II are not reviewed here since NBS only relies on newborn samples.

Recently, Stapleton et al. retrospectively analyzed 18,222 newborn DBS from the general population for IDUA and IDA activity and internal disaccharide biomarkers. The study identified a false positive rate of 0.29% for the enzyme activity assays alone. When all positives identified via the enzyme assay were subjected to a second-tier internal disaccharide screening, all false positives were eliminated, and one true positive was isolated. The fold differences above cutoff values for this MPS-II newborn were 1.13-fold for D0A0, 1.57-fold for D0S0, and 0.35-fold for D0a4 (i.e., overlap with the reference range) [24]. The clinical status and phenotype for the MPS-II patient identified in this study are not provided.

Consistent with literature reports, the dermatan sulfate D0a4 biomarker shows less elevation above the reference range than the heparan-sulfate-derived D0A0 and D0S0. Comparatively, D0a4 levels in MPS-I newborn DBS are slightly more elevated, with a minimum differential of 1.5-fold for severe MPS-I newborn DBS [16]. One possible explanation for this difference between MPS-I and MPS-II D0a4 levels is that 2-O-sulfated iduronic acid residues occur in dermatan sulfate in relatively low proportions compared to non-sulfated iduronic acid residues [30]. Due to the relatively lower occurrence of iduronic acid 2-O-sulfation (which accumulate in MPS-II) compared to overall iduronic acid residues (which accumulate in MPS-I), it is logical that the overall accumulation of dermatan sulfate in MPS-II patients would be lower than in MPS-I patients. This may explain why we see some elevation of the D0a4 dermatan sulfate biomarker in MPS-II newborn DBS, but the elevation is not as significant as that seen in MPS-I newborn DBS (see Figure 4).

The endogenous disaccharide method shows greater minimum differentials between random newborns and MPS-II newborn DBS than the internal disaccharide method (note that two MPS-II DBS samples were excluded, see below). The minimum differentials for severe MPS-II newborn DBS are 4.28-fold for endogenous disaccharide, UA-HNAc(1S)-late, and 2.40-fold for the endogenous trisaccharide, UA-HNAc-UA(1S). The minimum differentials between IDS pseudodeficiency newborn DBS and MPS-II newborn DBS were 1.77-fold and 1.43-fold, respectively. These fold differences do not include a single random newborn control with elevated endogenous biomarkers (Figure 7). Due to uncertainties about the stability of the MPS-II endogenous biomarkers, two MPS-II DBS samples stored at ambient conditions for 7.8 years (NY-610) and 14 years (NY-615) were not included in the minimum differential calculations (minimum differentials for UA-HNAc-UA(1S) were 1.38-fold and 0.00-fold, respectively).

**Figure 9:**
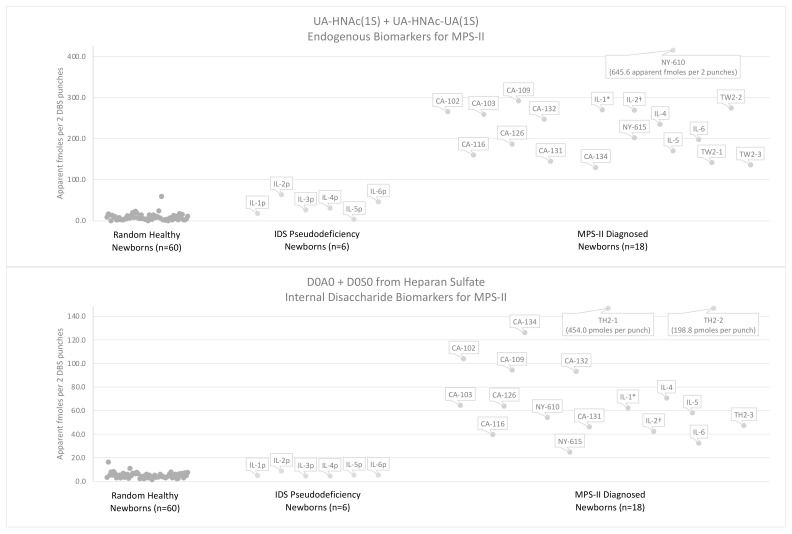
Levels of the MPS-II biomarkers from the internal disaccharide method (D0A0 and D0S0) and the endogenous biomarker method (UA-HNAc(1S)-late and UA-HNAc-UA(1S)) are added in newborn DBS from 60 random newborns (non-MPS), from 6 newborns with IDS pseudodeficiencies, from 16 newborns with severe MPS-II, from 1 newborn with undetermined MPS-II phenotype, and from 1 newborn with attenuated MPS-II phenotype. * DBS from patient with undetermined phenotype, † DBS from patient with attenuated phenotype.

Of the GAG-derived biomarkers investigated in this study, the endogenous disaccharide biomarker, UA-HNAc(1S)-late, shows the greatest discrimination between MPS-II and random newborn controls (minimum differential 4.28-fold). However, biomarker levels measured in the same method, such as levels of the heparan-sulfate-derived D0A0 and D0S0, are often combined to reduce false positive rates [29]. Figure 9 shows combined biomarker levels from the endogenous biomarker method (sum of UA-HNAc(1S)-late and UA-HNAc-UA(1S)) and the internal disaccharide method (sum of D0A0 and D0S0). The minimum differentials between random controls and newborn MPS-II DBS are 5.41-fold for the combined endogenous biomarkers and 3.01-fold for the combined internal disaccharides. The minimum differentials between the six IDS pseudodeficiency newborn DBS and MPS-II newborn DBS are 2.06-fold and 3.77-fold, respectively. When the levels of MPS-II internal disaccharide biomarkers are combined for random controls and patient samples, this method becomes more comparable to the endogenous biomarker method, though the internal disaccharide method shows greater discrimination between IDS pseudodeficiency newborn DBS and MPS-II newborn DBS. 

Data described in this report are limited to newborn MPS-II DBS from male patients. Although it is rare, MPS-II diagnoses have been reported in female patients. Reports of female MPS-II patients suggest that the accumulation of GAGs in diagnosed female patients is comparable to that in diagnosed male patients. In reports by Sukegawa et al., two instances of brother–sister siblings diagnosed with MPS-II demonstrate that the extent of elevations of urinary total GAGs, urinary heparan sulfate, and urinary dermatan sulfate are significant regardless of gender [6,7]. To date, there are no data available in the literature that report GAG levels in DBS from female MPS-II patients, and we were unable to obtain samples to include in this study. Published analyses of GAGs in female MPS-II patients include the measurement of total urinary GAGs by nonspecific methods relying on spectrophotometry, including exposure to carbazole [31], staining with Alcian Blue [32], or staining with 1,9-dimethylmethylene blue (DMB) [33]. The evaluation of urinary heparan sulfate and dermatan sulfate was performed by one-dimensional electrophoresis [34].

Table S8 summarizes urinary GAG measurements reported in the literature for female patients diagnosed with MPS-II and compares them to fold differences in GAG biomarkers measured in MPS-II and IDS pseudodeficiency DBS from newborn, male patients reported here. These data are reported for female patients between the ages of 2 and 12 whose total urinary GAGs were quantified by various methods (described above and in Table S8) and compared to age-corrected reference ranges. For these female patients, the fold difference from the normal range cutoff for total urinary GAGs ranged between 2.47-fold and 5.31-fold [4,6,7,8,9,10]. Sukegawa et al. report total urinary GAGs measured via the carbazole method in a pair of male–female siblings diagnosed with MPS-II, and the fold differences from the normal range were 4.07-fold for the female sibling and 4.62-fold for the male [7]. Data concerning levels of internal disaccharide and endogenous GAG biomarkers are still needed for MPS-II-diagnosed female newborn DBS. However, the consistent elevation of urinary GAGs in these six cases suggests that the GAG biomarker methods would likely be successful in identifying DBS from female newborn MPS-II patients in addition to males.

Our current analysis indicates that the endogenous biomarker method shows greater disease/non-disease discrimination than the internal disaccharide method for MPS-II newborn DBS. As is characteristic of the endogenous biomarker method, the measured response of the biomarkers is essentially background noise in random newborns (signal-to-noise is ~≤1, see Figure 5 and Figure 6). By comparison, the internal disaccharide biomarkers appear as distinguishable peaks (signal-to-noise ratio > 100) in random newborn controls (provided in Figure S2). Despite this difference, responses of the MPS-II endogenous biomarkers in patient DBS are weaker than the internal disaccharide biomarkers (endogenous signal-to-noise ratios are ~5–10). For this reason, the minimum differentials are comparable for the endogenous MPS-II biomarkers and the heparan sulfate internal disaccharide biomarkers, D0A0 and D0S0. 

## 5. Conclusions

The present study focused on MPS-II, which has been added to the screening protocols of several NBS laboratories. Data presented above indicate that, for MPS-II second-tier screening, the classic internal disaccharide method is comparably sensitive to the endogenous biomarker method. Previous evidence indicates that the inclusion of a second-tier GAG biomarker screening dramatically decreases false positives from first-tier enzyme assay screening. Based on this, we strongly recommend that either the internal disaccharide method or the endogenous biomarker method are included as a part of NBS protocols for MPS-II. It should be noted, however, that in both MPS-II (in this study) and in MPS I (in previous publications), only a limited number of samples from patients with attenuated disease have been available for analysis. Therefore, it cannot be stated with confidence that all affected patients will have elevated levels of the GAG biomarkers at birth. If this is not the case, the benefits of reducing false positives through the use of second-tier testing will have to be weighed against the disadvantage of potential missed cases.

## Figures and Tables

**Table 1:** Comparison of two glycosaminoglycan (GAG) analysis methods.

Method	Biomarkers	Sample	Sample Preparation	Internal Standard	Analysis
Internal Disaccharides	D0A0, D0S0, D0a4	1 × 3 mm DBS Punch	Enzymatic digestion with bacterial lyases	Chondrosine	Hypercarb column in reverse-phase, Xevo TQ-S Mass Spectrometer
Endogenous Biomarkers	UA-HNAc(1S)-late UA-HNAc-UA(1S)	2 × 3 mm DBS Punches	Derivatization with PMP	PMP-derivatized ∆UA-GalNAc(4S) ^1^	PFP column in reverse-phase, AB Sciex TQ 6500 ^2^

^1^ ΔUA-GalNAc(4S) is the same as D0a4 (see Table S3) and does not occur endogenously. ^2^ Alternatively, analytes may be detected using a Xevo TQ-S mass spectrometer.

## Data Availability

Data is contained within the article or supplementary material.

## Supplementary Materials

The following are available online at https://www.mdpi.com/article/10.3390/ijns8010009/s1.
