# Supplementary material for: Evaluation of Two Methods for Quantification of Glycosaminoglycan Biomarkers in Newborn Dried Blood Spots from Patients with Severe and Attenuated Mucopolysaccharidosis Type II
Source: Int J Neonatal Screen. 2022 Jan 21;8(1):9. doi: 10.3390/ijns8010009 (PMC8884011; doi:10.3390/ijns8010009)
Supplement: Supplementary file 1 [file IJNS-08-00009-s001.zip › IJNS-1525481-supplementary.pdf]

Supplementary Material

Table S1. Patient information provided by parents on 18 MPS-II patients. All data provided is copied verbatim from questionnaires filled by patients' parents. All patients presented are male.

ID*	Age at Diagnosis	Age at 1st Symptoms	1st Symptoms	Current Age	Current Symptoms	Genotype	Notes
CA-102	3.7yr	3yr	hand joint stiffness, language delay	10.9yr	limit range of major joint motion, develop delay, severe hip necrosis, facial puffiness	p.W502G (c.1504T>G)	Stored 10.9yr Frozen (-20ºC)
CA-103	1yr	1yr	Barely any symptoms; diagnosed after older sib. diagnosis, but did have big head circumf.	8.1yr	develop delay (mild/borderline normal), mild range of motion, hear loss, myopia	p.W502G (c.1504T>G)	Stored 8.1yr Frozen (-20ºC)
CA-109	3.2yr	3.2yr	late walker (18mo), crooked walk, falling a lot, clicking fingers	21.1yr	bed bound total care, tube fed, tracheostomy, 3L O2 room air	Not obtained	Stored 21.1yr Frozen (-20ºC)
CA-116	1.6yr	1.2yr	macrocephaly, hepatosplenomegaly	2yr	macrocephaly, hepatosplenomegaly, hearing loss, speech delay, freq. loose stools, freq. diarrhea, stiff/curved fingers	IDS, Exon 6, c.838_842del (p.Asn280Gln	Stored 2yr Frozen (-20ºC)

**Label key: CA = California*

Table S1. *(continued)*

ID*	Age at Diagnosis	Age at 1st Symptoms	1st Symptoms	Current Age	Current Symptoms	Genotype	Notes
CA-126	Prenatal	n/a	No symptoms noted as of date	1.6yr	Not provided	c.238C>T(p.Gln80*)	Stored 1.6yr Frozen (-20ºC); Received ERT at 1 month of age and stem cell transplant at 4 months of age
CA-131	1yr	0.7yr	Cardiac malformation (“hole in heart”), Abnormal hearing and speech, Clubbing of fingers	Deceased	Not provided	Not obtained	Stored Frozen (-20ºC)
CA-132	2.2yr	1.3yr	Umbilical and inguinal hernia, Chronic runny nose, Stiff joints, Frequent ear infections, Speech delay, Toe walking	6.3yr	Stiff joints, Frequent ear infections, Cognitive impairment, Speech delay, Hyperactivity, Hearing loss, Carpal tunnel syndrome	c.1018G>C	Stored 6.3yr Frozen (-20ºC)
CA-134	Not provided	2yr	Asthma	15.5yr	Not Provided	Not provided	Stored 15.5yr Frozen (-20ºC)

**Label key: CA = California*

Table S1. *(continued)*

ID*	Age at Diagnosis	Age at 1st Symptoms	1st Symptoms	Current Age	Current Symptoms	Genotype	Notes
NY-610	1.5yr	1.5yr	Family history	7.8yr	Sleep apnea, Hernias, Carpal tunnel, Short Stature, Ear tubes, Thickening of heart wall, MPS facial features, Cognitive delay, Joint stiffness, Curved fingers, Required tonsil adenoid surgery, Current enlarged adenoids	"Splice"	Stored 7.8yr ambient temp.
NY-615	1.5yr	1.5yr	Delayed milestones	14yr	Trouble walking, Requires hearing aids, Cognitive below age, Wears diapers	Never Obtained	Stored 14yr ambient temp.
TW2-1	Not provided	Not provided	Not provided	Not provided	Not provided	c.817C>T; p.R273W	Stored Frozen (-20ºC)
TW2-2	Not provided	Not provided	Not provided	3yr	Not provided	c.1025A>G, p.H342R	Stored Frozen (-20ºC)
TW2-3	Not provided	Not provided	Not provided	2yr	Not provided	c.311A>T, p.D104V	Stored Frozen (-20ºC)

**Label key: NY = New York, TW = Taiwan.*

Table S1. *(continued)*

ID*	Age at Diagnosis	Age at 1st Symptoms	1st Symptoms	Current Age	Current Symptoms	Genotype	Notes
IL-1	3 weeks	None	None	0.9yr	None	c.688A>T p.Ile230Phe	Stored 0.9yr Frozen (-20ºC); Undetermined phenotype
IL-2	2 weeks	None	None	1yr	None	c.897C>G p.S299R	Stored 1yr Frozen (-20ºC); Confirmed attenuated phenotype
IL-4	4 weeks	13 months	Macrocephaly	1.8yr	Macrocephaly, coarse facies, macroglossia, developmental delay	c.257C>T p.Pro86Leu	Stored 1.8yr Frozen (-20ºC) Confirmed neuronopathic phenotype
IL-5	3 weeks	2 years	Speech delay	2.2yr	Developmental delay	c.1506G>A p.Trp502*	Stored 2.2yr Frozen (-20ºC) Confirmed neuronopathic phenotype
IL-6	4 weeks	2 years	Speech delay	3.5yr	Developmental delay, hyperactivity	c.1403G>A p.R468Q	Stored 3.5yr Frozen (-20ºC) Confirmed neuronopathic phenotype

**Label key: IL = Illinois.*

Table S2. Patient information provided by parents on 6 IDS pseudodeficiency patients. All patients presented are male and are asymptomatic.

ID*	Age at Diagnosis	Current Age	Genotype	Plasma IDS (Cutoff: >155 nmol/4h/mL)**	Total Urinary GAGs*	Urinary Heparan Sulfate*	Urinary Dermatan Sulfate*	Notes
					(mg/mmol creatinine)**			
IL-1p	No Diagnosis	1.6yr	c.1499C>T	93.0	33.8	4.2	13.4	Stored 1.6yr Frozen (-20ºC)
IL-2p	No Diagnosis	1.4yr	c.1477C>T	5.1	31.8	44.2 (á)	23.8 (á)	Stored 1.4yr Frozen (-20ºC)
IL-3p	No Diagnosis	1.4yr	c.310G>A	6.6	28.3	18.4 (á)	18.5	Stored 1.4yr Frozen (-20ºC)
IL-4p	No Diagnosis	1.5yr	No Variant Identified	39.3	19.1	9.5 (á)	9.1	Stored 1.5yr Frozen (-20ºC)
IL-5p	No Diagnosis	1.1yr	Not provided	86.1	18.2	2.3	11.0	Stored 1.1yr Frozen (-20ºC)
IL-6p	No Diagnosis	3.5yr	c.1055A>G	5.3	15.5	13.1 (á)	8.4	Stored 3.5yr Frozen (-20ºC)

**Label key: IL = Illinois, "p" = Pseudodeficiencies, "(á)" = elevation above normal range.*

**IDS and GAG measurements performed at Greenwood Genetic Center. Total GAGs measured spectrophotometrically after treatment with 1,9-dimethylene blue (DMB). Heparan Sulfate and Dermatan Sulfate quantified by LC-MS/MS. Normal range for Total Urinary GAGs is 0-53.0 mg/mmol creatinine; Heparan Sulfate normal range is 0-5.3 g/mmol creatinine; Dermatan Sulfate normal range is 0-18.5 g/mmol creatinine.

Table S3. MPS-I patient information provided by parents on 4 new MPS-I patients. All data provided is copied verbatim from questionnaires filled by patients' parents. Additional MPS-I patients previously published (Herbst et. al. 2020).

ID*	Sex	Age at Diagnosis	Age at 1st Symptoms	1st Symptoms	Current Age	Current Symptoms	Genotype	Notes
CA-129	F	0.7yr	0.4yr	Congestion, large head	14.8yr	Pain, musculoskeletal deformities, short stature, short neck, corneal clouding	Not known	Stored 14.8yr Frozen (-20ºC)
TW1-1	Not provided	Not provided	n/a	No recorded symptoms	Not provided	No recorded symptoms	c.1874A>C, p.Y625S; c.300-3C>G	Stored Frozen (-20ºC)
TW1-2	F	3.2yr	1-2yr	Not provided	4yr	Not provided	c.1037T>G, p.L346R; c.1091C>T, p.T364M	Stored Frozen (-20ºC); Twin of TW1-3
TW1-3	F	1.6yr	1-2yr	Not provided	4yr	Not provided	c.1037T>G, p.L346R; c.1091C>T, p.T364M	Stored Frozen (-20ºC); Twin of TW1-2

**Label key: CA = California, TW= Taiwan.*

Table S4. GAG-derived saccharides related to MPS-II and their names.

**Compound**	**Structure**	**Name 1 (Lawrence et al., 2008)**	**Name 2 (Stapleton et. al, 2020)**	**Assay**
Dermatan sulfate/Chondroitin sulfate B internal disaccharide	ΔUA-GALNAc(4S)	D0a4	Chondro ΔDi-4S	Internal Disaccharide
Heparan sulfate internal disaccharide	ΔUA-GalNAc	D0A0	HS-0S	Internal Disaccharide
Heparan sulfate internal disaccharide	ΔUA-GalNS	D0S0	HS-NS	Internal Disaccharide
Heparan sulfate non-reducing end disaccharide	UA-HNAc(1S)	n/a	n/a	Endogenous Biomarker
Heparan sulfate non-reducing end trisaccharide	UA-HNAc-UA(1S)	n/a	n/a	Endogenous Biomarker

Figure S1. Liquid chromatography tandem mass spectrometry (LC-MS/MS) traces (multiple reaction monitoring (MRM) signal response versus time) for the MPS-II endogenous disaccharide UA-HNAc(1S) and the endogenous trisaccharide UA-HNAc-UA(1S) markers' 1-phenyl-3-methyl-5-pyrazolone (PMP) derivatives in newborn urine and fibroblasts from indicated patients.

(a) UA-HNAc(1S), late retention time in MPS-I Urine

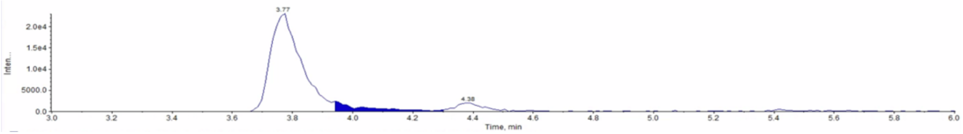

(b) UA-HNAc(1S), late retention time in MPS-II Urine

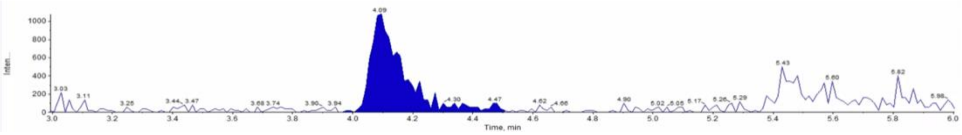

(c) UA-HNAc(1S), late retention time in MPS-I Fibroblasts

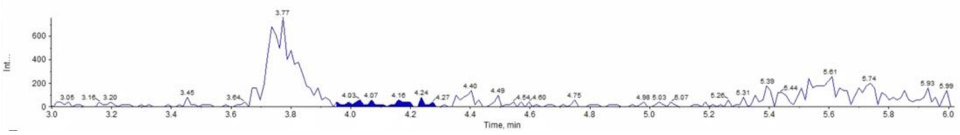

(d) UA-HNAc(1S), late retention time in MPS-II Fibroblasts

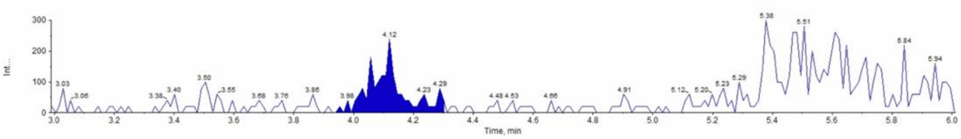

(e) UA-HNAc-UA(1S) in MPS-I Urine

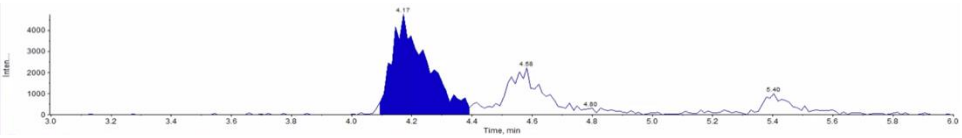

(f) UA-HNAc-UA(1S) in MPS-II Urine

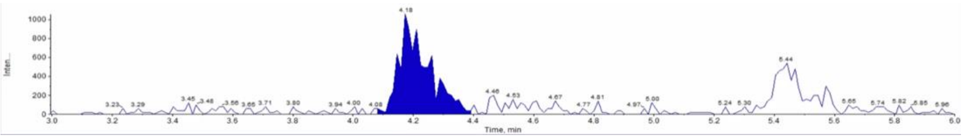

(g) UA-HNAc-UA(1S) in MPS-I Fibroblasts

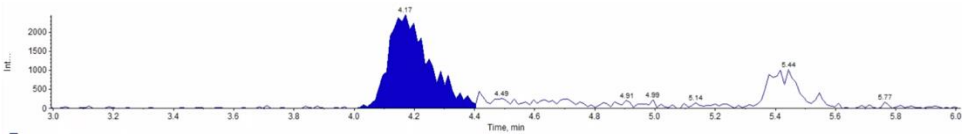

(h) UA-HNAc-UA(1S) in MPS-II Fibroblasts

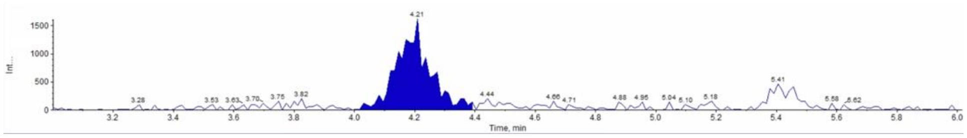

Table S5. Endogenous disaccharide marker (UA-HNAc(1S)) in MPS urine and fibroblasts. Shown are apparent μmole per mole creatinine (urine) and apparent fmole per mg protein (fibroblasts).

Sample Information	Urine (apparent μmoles/mole creatinine)	
	UA-HNAc(1S), late retention time	UA-HNAc-UA(1S)
Healthy	1	0
MPS-I	44	102
MPS-II	19	16
MPS-IIIA	4	1
MPS-IIIB	2	0
MPS-IIIC	1	1
MPS-IIID	9	1
MPS-IVA	2	1
MPS-IVB	2	2
MPS-VI	8	0
MPS-VII	5	9

Sample Information	Fibroblasts (apparent fmoles/mg protein)	
	UA-HNAc(1S), late retention time	UA-HNAc-UA(1S)
Healthy	n/a	n/a
MPS-I (*GM00798)	265	11,222
MPS-II (*GM01929)	631	5,444
MPS-IIIA (*GM00312)	130	1,626
MPS-IIIB (*GM00156)	129	707
MPS-IIIC	n/a	n/a
MPS-IIID	n/a	n/a
MPS-IVA (*GM00593)	138	1,112
MPS-IVB	n/a	n/a
MPS-VI (*GM00538)	158	2,462
MPS-VII (*GM02784)	47	225

**GM numbers for fibroblasts are the sample numbers from the Coriell Institute Cell Repository*

Table S6. Table of biomarker levels.

Sample Information			Newborn Dried Blood Spots (DBS)					
			UA-HNAc(1S)-Late	UA-HNAc-UA(1S)	D0A0	D0S0	D0a4 (Chondroitinase B)	D0a4 (Chondroitinase ABC)
			(apparent fmoles per two DBS punches)		(apparent pmoles per one DBS punch)			
MPS-II	Severe	CA-102	174	92	15.7	5.1	7.1	61.4
MPS-II	Severe	CA-103	169	91	11.3	1.6	7.9	47.8
MPS-II	Severe	CA-109	228	65	15.8	3.0	6.0	48.5
MPS-II	Severe	CA-116	104	56	6.8	1.1	2.1	29.7
MPS-II	Severe	CA-126	128	59	11.0	1.8	3.7	53.6
MPS-II	Severe	CA-131	91	54	8.0	1.3	2.5	33.6
MPS-II	Severe	CA-132	150	96	15.8	2.8	5.8	90.0
MPS-II	Severe	CA-134	92	38	21.6	3.6	1.7	73.7
MPS-II	Severe	NY-610	646	0	9.2	1.6	3.1	10.7
MPS-II	Severe	NY-615	188	14	3.6	1.3	1.4	14.1
MPS-II	Undetermined	IL-1	213	57	9.9	2.6	6.8	29.5
MPS-II	Attenuated	IL-2	201	68	6.6	1.9	1.4	27.1
MPS-II	Severe	IL-4	206	29	11.3	2.8	4.5	74.3
MPS-II	Severe	IL-5	146	25	9.9	1.8	*	41.5
MPS-II	Severe	IL-6	153	44	5.7	0.8	2.2	72.3
MPS-II	Severe	TW2-1	108	33	52.0	39.0	*	76.1
MPS-II	Severe	TW2-2	156	119	28.0	12.0	*	82.0
MPS-II	Severe	TW2-3	92	44	7.8	1.7	*	55.5
IDS Pseudodeficiency		IL-1p	17	0	0.8	0.2	0.4	12.9
IDS Pseudodeficiency		IL-2p	51	12	1.4	0.3	0.5	21.2
IDS Pseudodeficiency		IL-3p	23	3	0.8	0.2	0.5	20.0
IDS Pseudodeficiency		IL-4p	24	7	0.8	0.2	0.5	18.3
IDS Pseudodeficiency		IL-5p	0	3	0.9	0.2	0.3	23.9
IDS Pseudodeficiency		IL-6p	28	17	0.8	0.2	0.4	18.4

Table S6 *(continued)***.** Table of biomarker levels.

Sample Information	Newborn Dried Blood Spots (DBS)					
	UA-HNAc(1S)-Late	UA-HNAc-UA(1S)	D0A0	D0S0	D0a4 (Chondroitinase B)	D0a4 (Chondroitinase ABC)
	(apparent fmoles per two DBS punches)		(apparent pmoles per one DBS punch)			
Random Newborn (non-MPS)	13	10	1.0	0.2	0.6	15.9
Random Newborn (non-MPS)	16	2	1.3	0.2	1.1	24.3
Random Newborn (non-MPS)	16	0	2.1	1.2	0.8	19.6
Random Newborn (non-MPS)	8	1	1.3	0.4	1.0	35.8
Random Newborn (non-MPS)	8	0	1.2	0.2	0.7	23.1
Random Newborn (non-MPS)	7	0	0.9	0.1	0.8	29.2
Random Newborn (non-MPS)	2	6	1.2	0.3	0.7	38.0
Random Newborn (non-MPS)	5	0	0.6	0.1	0.8	23.4
Random Newborn (non-MPS)	14	6	1.1	0.2	0.6	29.0
Random Newborn (non-MPS)	6	1	1.1	0.2	0.9	31.2
Random Newborn (non-MPS)	10	4	1.0	0.1	0.4	21.2
Random Newborn (non-MPS)	6	1	1.1	0.1	1.0	20.7
Random Newborn (non-MPS)	8	4	1.3	0.2	1.4	22.9
Random Newborn (non-MPS)	11	0	1.0	0.2	1.8	31.3
Random Newborn (non-MPS)	13	0	1.0	0.1	0.8	26.2
Random Newborn (non-MPS)	41	18	0.9	0.1	0.8	27.5
Random Newborn (non-MPS)	14	0	0.6	0.2	0.4	64.9
Random Newborn (non-MPS)	12	0	0.8	0.2	0.6	23.1
Random Newborn (non-MPS)	8	1	0.9	0.3	1.4	46.5
Random Newborn (non-MPS)	6	0	1.1	0.2	0.5	23.8
Random Newborn (non-MPS)	12	3	1.2	0.2	1.5	32.6
Random Newborn (non-MPS)	13	0	0.9	0.2	0.5	28.5
Random Newborn (non-MPS)	1	0	0.6	0.1	0.8	11.8
Random Newborn (non-MPS)	8	2	0.4	0.1	1.3	16.3

Table S6 *(continued)***.** Table of biomarker levels.

Sample Information	Newborn Dried Blood Spots (DBS)					
	UA-HNAc(1S)-Late	UA-HNAc-UA(1S)	D0A0	D0S0	D0a4 (Chondroitinase B)	D0a4 (Chondroitinase ABC)
	(apparent fmoles per two DBS punches)		(apparent pmoles per one DBS punch)			
Random Newborn (non-MPS)	2	2	1.0	0.2	1.6	19.8
Random Newborn (non-MPS)	8	9	1.0	0.3	1.1	36.7
Random Newborn (non-MPS)	1	2	0.8	0.2	0.8	12.5
Random Newborn (non-MPS)	6	0	0.9	0.2	0.5	24.5
Random Newborn (non-MPS)	2	0	0.8	0.2	0.9	28.5
Random Newborn (non-MPS)	21	3	0.6	0.1	2.5	49.9
Random Newborn (non-MPS)	2	0	0.3	0.4	n/a	n/a
Random Newborn (non-MPS)	0	0	1.0	0.6	n/a	n/a
Random Newborn (non-MPS)	11	2	0.8	0.2	n/a	n/a
Random Newborn (non-MPS)	2	0	0.8	0.3	n/a	n/a
Random Newborn (non-MPS)	2	0	0.5	0.1	n/a	n/a
Random Newborn (non-MPS)	3	0	0.6	0.1	n/a	n/a
Random Newborn (non-MPS)	0	2	0.5	0.1	n/a	n/a
Random Newborn (non-MPS)	0	0	0.5	0.4	n/a	n/a
Random Newborn (non-MPS)	5	0	0.3	0.1	n/a	n/a
Random Newborn (non-MPS)	8	0	0.3	0.3	n/a	n/a
Random Newborn (non-MPS)	3	0	0.4	0.2	n/a	n/a
Random Newborn (non-MPS)	6	0	0.4	0.1	n/a	n/a
Random Newborn (non-MPS)	1	0	0.4	0.2	n/a	n/a
Random Newborn (non-MPS)	0	0	0.4	0.2	n/a	n/a
Random Newborn (non-MPS)	7	0	0.6	0.2	n/a	n/a
Random Newborn (non-MPS)	2	0	1.2	0.2	n/a	n/a
Random Newborn (non-MPS)	11	2	0.6	0.5	n/a	n/a
Random Newborn (non-MPS)	14	0	0.7	0.1	n/a	n/a

Table S6 *(continued)***.** Table of biomarker levels.

Sample Information	Newborn Dried Blood Spots (DBS)					
	UA-HNAc(1S)-Late	UA-HNAc-UA(1S)	D0A0	D0S0	D0a4 (Chondroitinase B)	D0a4 (Chondroitinase ABC)
	(apparent fmoles per two DBS punches)		(apparent pmoles per one DBS punch)			
Random Newborn (non-MPS)	9	0	0.5	0.2	n/a	n/a
Random Newborn (non-MPS)	0	0	0.4	0.2	n/a	n/a
Random Newborn (non-MPS)	5	0	1.0	0.2	n/a	n/a
Random Newborn (non-MPS)	4	0	0.6	0.6	n/a	n/a
Random Newborn (non-MPS)	3	0	0.2	0.1	n/a	n/a
Random Newborn (non-MPS)	4	0	0.4	0.1	n/a	n/a
Random Newborn (non-MPS)	1	1	1.2	0.1	n/a	n/a
Random Newborn (non-MPS)	6	0	1.9	0.3	n/a	n/a
Random Newborn (non-MPS)	2	0	0.4	0.1	n/a	n/a
Random Newborn (non-MPS)	3	1	0.6	0.3	n/a	n/a
Random Newborn (non-MPS)	4	0	0.5	0.1	n/a	n/a
Random Newborn (non-MPS)	7	1	0.3	0.4	n/a	n/a

Figure S2. Liquid chromatography tandem mass spectrometry (LC-MS/MS) traces (multiple reaction monitoring (MRM) signal response versus time) for the MPS-II internal disaccharide D0A0 and D0S0 and in newborn DBS from indicated patients digested with heparinases I, II, and III in buffer.

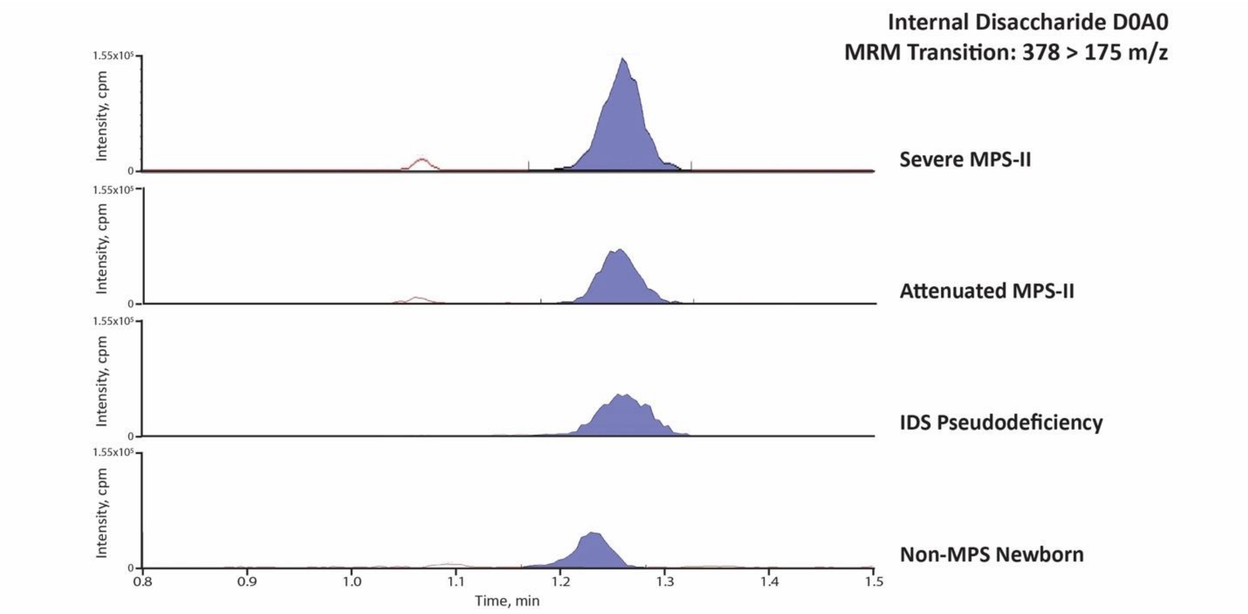

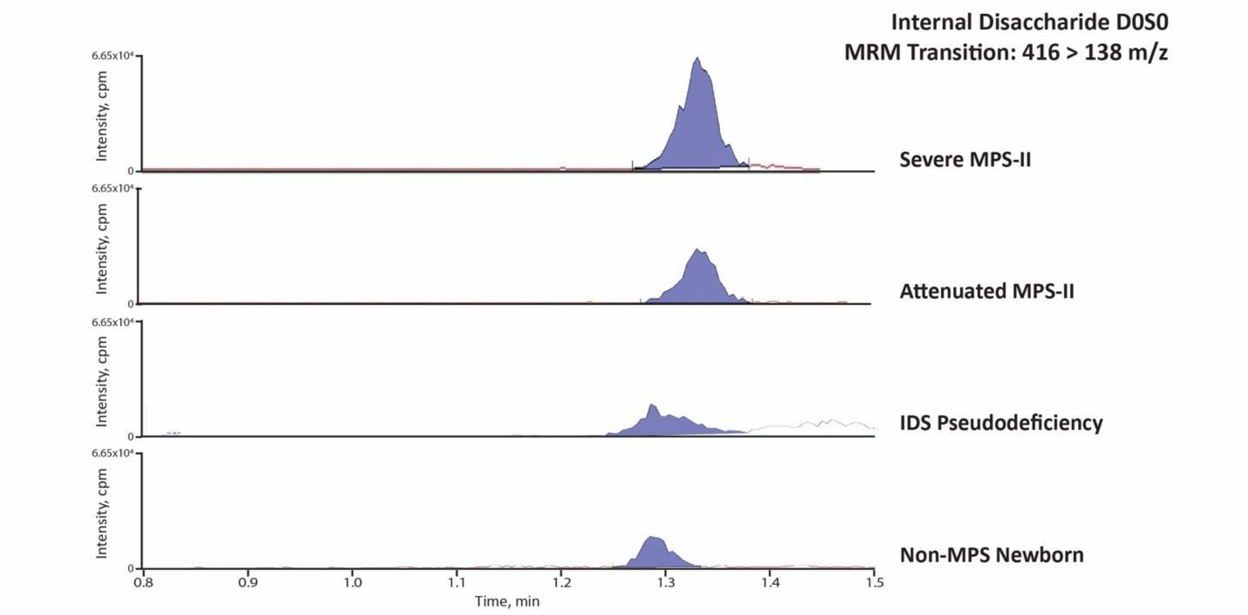

Table S7. Internal disaccharide markers in MPS urine. Shown are pmoles per nmole creatinine with MS Standard Correction.

**Sample Information**	**Urine Treated with Chondroitinase B (pmoles/nmole creatinine)**		
	**D0a4**	**D0A0**	**D0S0**
Healthy	0	0	0
MPS-I	28	21	4
MPS-II	2	4	1
MPS-IIIA	2	62	13
MPS-IIIB	1	36	7
MPS-IIIC	1	40	8
MPS-IIID	1	33	10
MPS-IVA	2	1	0
MPS-IVB	3	1	0
MPS-VI	1	1	0
MPS-VII	4	10	2

**Sample Information**	**Urine Treated with Chondroitinase ABC (pmoles/nmole creatinine)**			
	**D0a4**	**D0a6**	**D0A0**	**D0S0**
Healthy	0	1	0	0
MPS-I	40	8	24	4
MPS-II	5	2	4	1
MPS-IIIA	10	10	62	14
MPS-IIIB	4	2	40	7
MPS-IIIC	6	5	45	9
MPS-IIID	4	4	33	10
MPS-IVA	4	13	1	0
MPS-IVB	6	4	1	0
MPS-VI	1	1	1	0
MPS-VII	4	5	13	2

Table S8. Fold differences in GAG levels between reference range cutoffs and a single patient sample for the following markers: added endogenous biomarkers in DBS (see Figure 8), added heparan sulfate internal disaccharide biomarkers in DBS (see Table 7), and total urinary GAGs (uGAGs) measured by various methods in from various published sources.

**Sample**	**Added DBS Endogenous Biomarkers**	**Added DBS Internal Disaccharides (D0A0 + D0S0)**	**Total Urinary GAGs (uGAG)**	**Method of uGAG Analysis***
	**Fold-Difference**	**Fold-Difference**	**Fold-Difference**	
MPS-II Male Newborns (Minimum)	5.41-Fold	3.01-Fold	n/a	**n/a**
MPS-II Male Newborns (Maximum)	12.19-Fold	11.62-Fold	n/a	**n/a**
IL-1p (IDS Pseudo. Male Newborn)	0.70-Fold	0.45-Fold	0.64-Fold**	**DMB**
IL-2p (IDS Pseudo. Male Newborn)	2.63-Fold	0.80-Fold	0.60-Fold**	**DMB**
IL-3p (IDS Pseudo. Male Newborn)	1.09-Fold	0.42-Fold	0.53-Fold**	**DMB**
IL-4p (IDS Pseudo. Male Newborn)	1.27-Fold	0.42-Fold	0.36-Fold**	**DMB**
IL-5p (IDS Pseudo. Male Newborn)	0.13-Fold	0.47-Fold	0.34-Fold**	**DMB**
IL-6p (IDS Pseudo. Male Newborn)	1.90-Fold	0.49-Fold	0.29-Fold**	**DMB**
Broadhead 1986, Female with MPS-II	n/a	n/a	5.25-Fold	**AB**
Sukegawa 1997, Female with MPS-II	n/a	n/a	4.30-Fold	**Carbazole**
Sukegawa 1998, Female Sibling with MPS-II	n/a	n/a	4.07-Fold	**Carbazole**
Sukegawa 1998, Male Sibling with MPS-II	n/a	n/a	4.62-Fold	**Carbazole**
Kloska 2011 & Jurecka 2012, Female with MPS-II	n/a	n/a	2.95-Fold	**Not reported**
Jurecka 2012, Female with MPS-II	n/a	n/a	5.31-Fold	**Not reported**
Lonardo 2014, Female with MPS-II	n/a	n/a	2.47-Fold	**DMB**

* Carbazole [Bitter 1962], Alcian Blue Staining (AB) [Whiteman 1973], 1,9-dimethylmethylene Blue Staining (DMB) [Coppa 1987].

** GAG measurements for IDS pseudodeficiency newborn DBS were performed at Greenwood Genetic Center

STANDARD OPERATING PROCEDURE:
ENDOGENOUS BIOMARKER METHOD FOR MPS-II SAMPLES

MPS Samples and Controls:

- Coriell Institute MPS Patient Fibroblasts: MPS-I (GM00798), MPS-II (GM01929), MPS-IIIA (GM00312), MPS-IVA (GM00593), MPS-VI (GM00538), MPS-VII (GM02784)
- Anonymous healthy adult patient urine sample
- De-identified MPS newborn patient urine samples dried on filter paper (from Dr. Maria Fuller, University of Adelaide, Australia): MPS-I, MPS-II, MPS-IIIA, MPS-IIIB, MPS-IIIC, MPS-IIID, MPS-IVA, MPS-IVB, MPS-VI, MPS-VII
- De-identified healthy newborn dried blood spots (DBS) (provided by Dr. Zoltan Lukacs, University of Hamburg, Germany)

Materials: Standards and Reagents.

- Internal Standard (IS), Chondroitin Disaccharide di-4S (CAS 136144-56-4) (Carbosynth, Ref: OC28898)
- PMP, 3-Methyl-1-phenyl-2-pyrazoline-5-one (CAS 89-25-8) (Aldrich, Ref: M70800)
- Ammonia Solution 28-30% (CAS 1336-21-6) (MilliporeSigma Ref: 105423)
- Methanol, HPLC Grade (CAS 67-56-1) (Fisher, Ref: A452-1)
- Formic Acid, Optima Grade (CAS 64-18-6) (Fisher, Ref:A117-50)
- Chloroform, HPLC Grade (CAS 67-66-3) (Fisher, Ref: C606-1)
- Acetonitrile, Optima Grade (CAS 75-05-8) (Fisher, Ref: A955-500)
- Water, Optima Grade (CAS 7732-18-5) (Fisher, Ref: W6500)
- 1x DPBS (ThermoFisher, Ref: 14190144)

Materials: Benchtop Supplies.

- Pierce™ BCA Protein Assay Kit (ThermoFisher, Ref: 23225)
- pH Test strips, 4.5-10.0 pH (MilliporeSigma, Ref: P4536)
- 3 mm paper hole puncher
- Microtubes with snap caps, 1.5 mL polypropylene (VWR, Ref: 89000-028)
- Thin-walled PCR tubes, 0.5 mL (Sigma Aldrich, Ref: CLS6530)
- Peltier Thermal Cycler, PTC-200 (MJ Research, Ref: 8252-30-0001)
- MilliQ Water Filtration System
- SpeedVac, Vacuum Concentrator
- Benchtop Centrifuge
- Sun-Sri assembled septum-capped vials (Thermo Scientific Ref: 200 428)

Materials: Mass Spectrometry.

- Pulled-point glass HPLC vial inserts, 250 µL (Agilent, Ref: 5183-2085)
- HPLC Vials, 2 mL (J.G. Finneran, Ref: 32009M-1232)
- HPLC Vial Cap, pre-slit septa (ThermoScientific, Ref: C5000-45B)
- Pursuit 3 PFP 2.0 x 100 mm 3 µm HPLC column (Agilent, Ref: A3051100x020)
- AB Sciex 6500 Mass Spectrometer with Water's I-Class Acquity UPLC with flow-through needle injector
- (alternatively) A Waters Xevo-TQ-S Mass Spectrometer is sensitive enough to detect GAG markers

Materials: Urinary Creatinine Measurement.

- Creatinine, anhydrous (CAS 60-27-5) (Sigma Aldrich, Ref: C4255)
- d3-Creatinine (CAS 143827-20-7) (Cayman Chemical, Ref: 16763)
- MicroTube Rack System, 0.65 mL polypropylene (Unifit Brand Tips, Ref: TN0933-01R)
- Waters Acquity UPLC BEH Shield RP18 1.7 µm column (Waters, Ref: 186002854)
- Waters Xevo-TQ Mass Spectrometer with Water's Acquity UPLC with fixed loop injector

Preparation of Fibroblast Samples:
Suspend pelleted fibroblasts in 1x PBS and lyse via freeze-thaw in -80°C freezer. Mix well and complete cell lysis via vortexing, centrifuge, and collect the supernatant in a new tube. Measure the protein content in the supernatant using the Pierce BCA assay kit from ThermoFisher (BSA standard). Transfer an aliquot containing 75 µg protein (MPS-I) or 105 µg protein (MPS-II) to a labeled 0.5 mL PCR tube and dry in a SpeedVac concentrator prior to derivatization with PMP.

Preparation of Urine Samples:
Determine the concentration of urinary creatinine using the LC-MSMS method described at the end of this SOP. Transfer an aliquot of the urine containing 20 nmol creatinine to a labeled 0.5 mL PCR tube and dry in a SpeedVac concentrator prior to derivatization with PMP.

Preparation of DBS Samples:
Make two 3 mm punches from each DBS spot and transfer them to a 1.5 mL capped microtube. Add 50 µL MilliQ water, cap, and incubate for 2 hours at 37°C while shaking at 250 rpm. Centrifuge briefly to collect all droplets of liquid at the bottom of the tube, then transfer liquid to a labeled 0.5 mL PCR tube. Dry the sample in a SpeedVac prior to PMP derivatization.

PMP Derivatization Method:

Prepare the following solutions:

- <u>0.2 mM Chondroitin disaccharide di-4S (IS) stock in water</u>: To 1 mg chondroitin disaccharide di-4S (503.34 g/mol), add 198.7 μL MilliQ water to make 10 mM solution. Dilute 10 μL of the 10 mM stock to 500 μL using MilliQ water to make 0.2 mM stock. Aliquot the 0.2 mM stock into vials with Teflon-septum screw caps and store all stocks at -20°C. Seal the vials with Parafilm to prevent evaporation.
- <u>2.0 M Formic Acid in water</u>: Dilute 377.3 μL Optima Grade formic acid (46.03 g/mol; 1.22 g/cm3) to 5 mL using MilliQ water.
- <u>0.4 M Ammonia in 24% Methanol in Water</u>: To make solution, mix 35.5 mL MilliQ water with 11.9 mL HPLC grade methanol. Add 1.65 mL of 28-30% NH3. Mix gently to combine and use within 1 week.
- <u>0.25 M PMP with 1.0 μM IS</u>: Weigh out 43.6 mg PMP and dissolve in 1.0 mL of 0.9 M ammonia solution. The solution may be heated up to 70°C to help dissolve PMP solids if necessary. Spike the solution with 5.0 uL of 0.2 mM chondroitin disaccharide di-4S ("IS"). Prepare this solution fresh before each derivatization reaction.

Start the PCR program to warm up the tray to 70ºC. To each dried sample in a 0.5 mL PCR tube, add 100 μL of the 0.25 M PMP solution with 1.0 μM IS. Mix the reaction tubes well via vortexing, then load them onto the PCR tray and start a timer for 90 min.

After 90 min, remove the samples from the PCR tray and allow them to cool for 10 minutes. Dilute the samples with 50 μL of 2.0 M formic acid in water to acidify, cap the tube, and vortex. Hold samples in a vial rack, then add 200 μL chloroform to wash PMP from the aqueous layer. Cap the tubes, cover the sample rack with a second empty rack to keep vials from falling out, and shake vigorously by hand for one minute. Centrifuge the samples for 1 minute at 13,000g to separate the layers (centrifuge longer if an emulsion has formed). Pre-wet a disposable Pipetman tip with clean chloroform and use it to remove the bottom chloroform layer from the sample for disposal. Reserve the aqueous layer in the tube. Wash the aqueous layer three more times (for a total of four times) with 200 μL chloroform, following the steps above.

After chloroform washing, the aqueous layer should be approximately 150 μL. For DBS samples, the aqueous layer should be further concentrated in a SpeedVac to a volume of approximately 70 μL. Urine and fibroblast samples do not need to be concentrated. Centrifuge the samples for 15 minutes at 3,000g, then transfer the aqueous sample to a 250 μL pulled-point glass insert in a 2 mL HPLC vial for analysis via LC-MS (described below).

LC-MSMS Analysis:

The derivatized glycosaminoglycan (GAG) markers were separated using a 2.0x100 mm 3 μm Pursuit 3 PFP column (Agilent P/N: A3051100X020) connected to a Waters I-Class Acquity UPLC with a direct infusion syringe pump. The column was held at room temperature and the markers were eluted with a 12-minute UPLC gradient program using 0.1% formic acid in water (MPA) and 0.1% formic acid in acetonitrile (MPB). A strong needle wash of 100% acetonitrile and a purge wash and seal wash of 100% water were used. All data was collected with an AB Sciex 6500 TQ mass spectrometer with an IonSpray source in negative mode (Xevo TQ-S may be used instead, see parameters below). Nitrogen was used as a curtain

gas at 30 psi, collision gas was 12 units, IonSpray voltage was -4500 V, source temperature was 400°C, and ion source gas 1 and gas 2 were each 40 units. See tables below for UPLC gradient program, MRM tuning information, and marker retention times.

UPLC Solvent Gradient Program

Time (min)	Flow (mL/min)	%MPA	%MPB
0.0	0.3	85	15
5.0	0.3	77.5	22.5
5.5	0.3	10	90
7.5	0.3	10	90
8.0	0.3	85	15
12.0	0.3	85	15

Marker Names, Retention Times, MRMs, and Tuning Information for AB Sciex 6500

Marker	Disease	Retention Time (RT)	MRM	DP	EP	CE	CXP
UA-GalNAc-4S	Internal Standard	3.6 min	788.1 > 534.1	-70	-10	-35	-25
UA-HNAc (1S) (early RT)	MPS-I	3.6 min	806.3 > 294.9	-84	-10	-45	-25
UA-HNAc (1S) (late RT)	MPS-II	4.1 min	806.3 > 294.9	-84	-10	-45	-25
UA-HNAc-UA (1S)	MPS-II	4.2 min	982.3 > 330.9	-87	-10	-41	-27
HN-UA (1S)	MPS-IIIA	4.3 min	764.2 > 331.1	-80	-10	-45	-25
(HNAc-UA)2(1S)	MPS-IIIB	3.9 min	1185.2 > 931.4	-80	-10	-45	-25
(HN-UA)2-HNAc (1S)	MPS-IIIC	3.6 min	691.8 > 605.0	-40	-10	-24	-25
HNAc (1S), "GlcNAc(6S)"	MPS-IIID	4.0 min	630.4 > 256.1	-80	-10	-45	-25
HNAc (1S), "GalNAc(6S)"	MPS-IVA	4.4 min	630.4 > 256.1	-80	-10	-45	-25
(Hex-HNAc)2 (2S)	MPS-IVB	3.1 min	1240.0 > 256.1	-87	-10	-41	-25
HNAc (1S), "GalNAc(4S)"	MPS-VI	4.5 min	630.4 > 256.1	-80	-10	-45	-25
UA-HN-UA (1S)	MPS-VII	4.1 min	940.0 > 331.1	-80	-10	-44	-25

(Alternative LC-MS/MS Option)

Xevo TQ-S Parameters

Parameter	Setting
Polarity	ESI-
Capillary (kV)	2.0
Source Temperature (^oC)	150
Desolvation Temperature (^oC)	600
Cone Gas Flow (L/hr)	150
Desolvation Gas Flow (L/hr)	900
Collision Gas Flow (mL/min)	ON
Collision Gas	Argon

(Alternative LC-MS/MS Option)

Marker Names, Retention Times, MRMs, and Tuning Information for Xevo TQ-S

Marker	Disease	Retention Time (RT)	MRM	Cone (V)	Collision
UA-GalNAc-4S	Internal Standard	4.8 min	788.1 > 534.1	42	24
UA-HNAc (1S) (early RT)	MPS-I	4.9 min	806.3 > 294.9	40	27
UA-HNAc (1S) (late RT)	MPS-II	5.3 min	806.3 > 294.9	40	27
HN-UA (1S)	MPS-IIIA	5.5 min	764.2 > 331.1	32	26
(HNAc-UA)2(1S)	MPS-IIIB	5.2 min	1185.2 > 931.4	30	32
(HN-UA)2-HNAc (1S)	MPS-IIIC	4.9 min	691.8 > 605.0	28	14
HNAc (1S), “GlcNAc(6S)”	MPS-IIID	5.2 min	630.4 > 256.1	24	28
HNAc (1S), “GalNAc(6S)”	MPS-IVA	5.6 min	630.4 > 256.1	24	28
(Hex-HNAc)2 (2S)	MPS-IVB	4.4 min	1240.0 > 256.1	28	36
HNAc (1S), “GalNAc(4S)”	MPS-VI	5.7 min	630.4 > 256.1	24	28
UA-HN-UA (1S)	MPS-VII	5.4 min	940.0 > 331.1	44	34

STANDARD OPERATING PROCEDURE:
INTERNAL DISACCHARIDE METHOD FOR MPS-II SAMPLES

MPS Samples and Controls:
- Donated anonymous healthy newborn dried blood spots (DBS) (from Dr. Francyne Kubaski, Universidade Federal do Rio Grande, Brazil and Dr. Zoltan Lukacs, University of Hamburg, Germany)
- Donated anonymous MPS-I newborn dried blood spots (DBS) (from the National MPS Society, United States)

Materials: Standards and Reagents.
- Internal Standard (IS), "Chondrosine" 2-Amino-2-deoxy-3-O-(b-D-glucopyranuronosyl)-D-galactopyranose (CAS 499-14-9) (Carbosynth, Ref: OA10113)
- Chondroitin/Dermatan Sulfate Disaccharide "D0a4" (CAS 136144-56-4) (Iduron, Ref: CD002)
- Chondroitin/Dermatan Sulfate Disaccharide "D0a6" (CAS 136132-72-4) (Iduron, Ref: CD003)
- Heparan Disaccharides Mixture "D0A0" and "D0S0," I mL of 75 nM (Iduron, Ref: HD Mix)
- Chondroitinase B from *Flavobacterium heparinum*, 50 UN (Sigma, Ref: C8058-50UN, 1UN* = 0.1 µmole/hr)
- Chondroitinase ABC from *Proteus vulgaris,* 2 IU (AMSBIO, Ref: AMS.E1028-02, 1IU* = 1.0 µmole/min)
- Heparinase I from *Flavobacterium heparinum*, 0.5 IU, lyophilized (Ibex, Ref: 60-400, 1IU* = 1.0 µmole/min)
- Heparinase II from *Flavobacterium heparinum*, 0.5 IU, lyophilized (Ibex, Ref: 60-400, 1IU* = 1.0 µmole/min)
- Heparinase III from *Flavobacterium heparinum*, 0.5 IU, lyophilized (Ibex, Ref: 60-400, 1IU* = 1.0 µmole/min)
- Bovine Serium Albumin (BSA) lyophilized powder (Sigma, Ref: A0281-10G)
- TRIS Ultra-Pure Grade (Amresco, Ref: 201-064-4)
- Dithiothreitol (DTT) (CAS 3483-12-3) (Sigma Aldrich, Ref: D0632)
- Calcium chloride (CAS 7440-70-2) (Sigma Aldrich, Ref: C-3306)
- Methanol, HPLC Grade (CAS 67-56-1) (Fisher, Ref: A452-1)
- Ammonia Solution 28-30% (CAS 1336-21-6) (MilliporeSigma Ref: 105423)
- Methanol, Optima Grade (CAS 67-56-1) (Fisher, Ref: A456-4)
- Water, Optima Grade (CAS 7732-18-5) (Fisher, Ref: W6500)

** 1.0 International Unit (IU) = 600 "Sigma" Units (UN) (check definition of units on company spec sheets to be sure)*

Materials: Benchtop Supplies.
- Deep-well 96-well plate, polypropylene, round bottom, 1mL well (Costar, Ref: 3959)
- Sealing film for 96-well plate (Axygen, Ref: PCRSP)
- MilliQ Water Filtration System
- N2 stream evaporator with 96-well plate adapter (made in-house)
- Allegra X-12R Centrifuge (Beckman Coulter)

Materials: Mass Spectrometry Supplies/Equipment.
- Shallow-well 96-well plate, polypropylene, v-bottom, 350 µL well (Greiner Bio-One, Ref: 651201)

- Hypercarb porous graphitic carbon column, 50 x 2.1 mm, 5 µm (Thermo, Ref: 35005-052130)
- Hypercarb guard columns, 10 x 2.1 mm, 5 µm (Thermo, Ref: 35005-012101)
- Universal uniguard pre-column holder (Thermo, Ref: 852-00)
- Waters Xevo TQ-S Mass Spectrometer with Water's I-Class Acquity UPLC with flow-through needle injector

Lyase Digestion of Samples:

Prepare the following solutions:

- 50 mM Tris Buffer, 11 mM Calcium Chloride, pH 7.0. Dissolve 181.7 mg Tris base and 48.5 mg calcium chloride dihydrate in 10 mL MilliQ water. Adjust the pH with HCl and bring volume to 30 mL with MilliQ water. Store at 4°C.
- 100 mM DTT stock in water: Dissolve 15 mg DTT per 1.00 mL of MilliQ water. Prepare fresh before each assay.
- 1% BSA in water: Dissolve 0.5 g BSA in 50 mL MilliQ water. Store at 4°C.
- 0.1 mM Chondrosine (IS) stock in water: Use qNMR to determine the exact amount of chondrosine you are starting with. For each 1 mg of chondrosine (355.3 g/mol), add 281.5 µL MilliQ water to make 10 mM solution. With a Hamilton Syringe, dilute 5 µL of the 10 mM stock to 500 µL using MilliQ water to make 0.1 mM stock. Aliquot the 0.1 mM stock into vials with Teflon-septum screw caps and store all stocks at -20°C. Seal the vials with Parafilm to prevent evaporation.
- 1 mg/mL (1.99 mM) D0a4 in water: Make 1 mg/mL stock by adding 1 mL MilliQ water to 1 mg of D0a4 powder. This is not quantified by qNMR.
- 1 mg/mL (1.99 mM) D0a6 in water: Make 1 mg/mL stock by adding 1 mL MilliQ water to 1 mg of D0a6 powder. This is not quantified by qNMR.
- 75 nM D0A0 and D0S0 stock: Use HD Mix stock as received from the vendor. This is not quantified by qNMR.
- MS Standard Solution: Prepare enough MS Standard solution for 200 response factor checks, or 2 mL. Prepare the standard by combining 20.0 µL of 10 mM Chondrosine (IS), 20.1 µL of 1 mg/mL (1.99 mM) D0a4, 20.1 µL of 1 mg/mL (1.99 mM) D0a6, 533.3 µL of HD Mix (75 nM), and 1,406.5 µL MilliQ water. Per each set of assays, 10 µL of this stock will be added to a blank well with 200 µL MilliQ water. Throughout the LC-MS/MS run, this MS Standard will be injected throughout the run to obtain a biomarker:chondrosine peak response factor for each analyte. This response factor is then used to correct biomarker measurements for each sample (see below).
- Heparinase I, II, and III stocks*: Dissolve Heparinase I, II, and III (0.5 IU) in 0.25 mL of 1% BSA in water. This is a final concentration of 2.0 IU/mL. Aliquot into 10 µL fractions and store at -20°C to avoid multiple freeze-thaw cycles. More dilute solutions may be made if less than 20 samples will be run at a time.
- Chondroitinase B stock*: Make 66.7 mIU/mL stock by diluting 83 mIU (50 UN) to 1244.4 µL using 1% BSA in MilliQ water. Aliquot into 10 µL fractions and store at -20°C to avoid multiple freeze-thaw cycles.
- Chondroitinase ABC stock*: Make 10 IU/mL stock by diluting 2 IU to 200 µL using 1% BSA in MilliQ water. Aliquot into 10 µL fractions and store at -20°C to avoid multiple freeze-thaw cycles.

** Enzymes seem to be stable with up to 3 freeze-thaw cycles, so make aliquots such that you can keep the number of cycles to 3, discarding after 3 cycles.*

NOTE: The biomarker:chondrosine response factor is calculated as described here. We do not assume that all biomarker's MSMS response factors are unity relative to chondrosine. That is, if you inject the same mole amounts of each disaccharide and chondrosine, the intensity of the disaccharides' MSMS response (peak area) will not be equal to that of chondrosine. Using the MS Standard, which is a solution containing known mole amounts of each disaccharide analyte and chondrosine, we can measure the difference in MSMS response for each analyte and chondrosine. The response factors are determined by injecting the MS Standard multiple times throughout every LC-MS/MS run. Response factor is calculated for each disaccharide biomarker by dividing the biomarker peak response by the chondrosine (IS) peak response. Then, all data measured during that run is divided by the response factor as a correction.

Punch one 3 mm DBS disk per patient and transfer the punches into a well of a 1 mL deep well 96-well plate (Costar Ref: 3959). For every 50 samples, combine the following reagents to prepare the lyase buffer:

4.5 mL of 50 mM Tris HCl with 11 mM $CaCl_2$, pH 7.0 (Amresco Ref: 0497)
150 µL of 100 mM DTT solution in water
500 µL of 0.1 mM Chondrosine (IS), 1 nmole per well

Next, prepare 1 mL of the lyase enzyme stock by combining the following enzymes and diluting with 1% BSA:

*5 µL of 10 IU/mL chondroitinase ABC, 1.0 mIU per well
*500 µL of 66.7 mIU/mL chondroitinase B, 0.667 mIU per well
25 µL of 2 IU/mL heparinase I, 1.0 mIU of per well
25 µL of 2 IU/mL heparinase II, 1.0 mIU of per well
25 µL of 2 IU/mL heparinase III, 1.0 mIU of per well
*Either chondroitinase B or chondroitinase ABC was used per sample, not both.

Vortex the lyase buffer prior to adding the lyase enzymes. Only mix the lyase enzymes by gently pipetting up and down. For 50 samples, the final volume of combined reagents was 6,150 µL. Add 123 µL of this combined lyase enzymes, lyase buffer, and internal standard to each well.

Seal the plate with sealing film and incubate at 37°C while shaking at 250 rpm. After overnight incubation (16h ± 1h), quench each sample with 400 µL of cold methanol, pipetting up and down 20 times to mix and precipitate proteins. Centrifuge the plate for 15 min at 3,000 *g*. Transfer 400 µL of supernatant in fractions to a new v-bottom 96-well microplate (Griener Bio-One Ref: 651201) and concentrate to dryness with N2 stream. Reconstitute samples in 100 µL water and mix up and down 20x with a pipette. Measure D0a4, D0a6, D0A0, and D0S0 levels via UPLC-MS/MS.

To check the relative response of markers to IS, prepare a blank well with 100 µL of water (no chondrosine or enzymes) and spike with 10 µL of MS Standard. Inject this standard into the MS prior to analyzing samples to ensure that there has been no drift in the response between the chondrosine (IS) and the markers (D0a4, D0a6, D0A0, D0S0) since the last assay. Use the same solution of MS Standard for each

assay so that the relative responses may be compared between assays. If a new solution of MS Standard must be made (when the old solution begins to run out), inject the old solution alongside the new solution so that the new solution's relative ratios can be verified before being used as a standard for future assays. Note, everything relates back to the chondrosine MSMS signal which is based on the qNMR-standardized stock solution of chondrosine IS. See the note above about response factor calculation.

The new levels of GAG markers will be measured relative to the response of 1 nmole chondrosine per 1 DBS punch, then will be divided by the biomarker:chondrosine response factor (determined from MS Standard injections). The response ratio of each marker from an MPS patient's DBS will be recorded and compared to the DBS of healthy patients in order to determine if the level of marker meets a cut-off level. These cut-off values will need to be re-determined if the method is transferred to another MS instrument.

Method for LC-MSMS Analysis:

Disaccharides were separated using a 5 μm 50 x 2.1mm Hypercarb porous graphitic carbon column (Thermo, P/N 35005-052130) fitted with a Hypercarb 5 μm 10 x 2.1mm guard column (Thermo, P/N 35005-012101) in a universal uniguard holder (Thermo, P/N 852-00) connected to a Waters I-Class Aquity UPLC with a flow-through needle. The column was held at 60ºC. The disaccharides were eluted in a 5-minute UPLC gradient program with 148 mM ammonia in water (MPA) and 100% acetonitrile (MPB). See the program in the table below. A strong needle wash of 100% acetonitrile, a purge wash of MPA, and a seal wash of 90/10 water/acetonitrile were used.

Using the MS-Standard:

The MS-Standard stock was prepared as described above, then 10 μL of this stock was diluted to 200 μL using MilliQ water. The MS-Standard contains 0.1 mM chondrosine, 0.02 mM D0a4, 0.02 mM D0a6, 0.02 mM D0S0, 0.02 mM D0A0, and 0.02 mM D0A6 (D0a6 and D0A6 biomarkers were not reported for MPS-II newborn patient DBS). From the MS-Standard was calculated the response factor for each analyte by dividing the MRM peak area for the analyte by the MRM peak area for chondrosine, then dividing by 0.2 (0.02 mM analyte / 0.1 mM chondrosine in the MS-Standard).

The MS-Standard is injected twice at the beginning of each run, then once every 15 samples, then once at the very end of the run. The response factor is calculated for each analyte (as above) using these MS-Standard injections. The %CV of response factors measured in all MS-Standard injections is then determined. If the %CV exceeds 20% for an analyte, the run fails the MS-Standard QC test and the data is not used. This QC test is done because the chondrosine internal standard is not chemically identical to any analytes, and any changes to ionization efficiency of the analyte may not be reflected in this internal standard. The MS-Standard measures if these differences become significant (through changes in the response factor) throughout the run.

The average response factor for each analyte was then determined using all MS-Standard injections. For every analyte measured in a patient sample using the internal disaccharide method, the apparent moles of the analyte (determined by dividing the MRM peak area of the analyte by the MRM peak area of the internal standard, chondrosine) is corrected by dividing apparent moles by the average response factor, then dividing by the number of DBS punches, or the amount of creatinine (for urine samples).

UPLC Solvent Gradient Program:

Time (min)	Flow (mL/min)	%MPA	%MPB
0.0	0.60	100	0
0.1	0.60	100	0
2.0	0.60	65	35
2.3	0.60	65	35
2.5	0.60	0	100
2.9	0.60	0	100
3.0	0.60	100	0
5.0	0.60	100	0

Disaccharides were detected with a Waters Xevo TQ-S mass spectrometer with an ESI source in negative mode. See mass spec parameters and MRMs below. Waters MassLynx Software and TargetLynx was used to analyze the data.

Xevo TQ-S Parameters

Parameter	Setting
Polarity	ESI-
Capillary (kV)	2.0
Source Temperature (^{o}C)	150
Desolvation Temperature (^{o}C)	550
Cone Gas Flow (L/hr)	150
Desolvation Gas Flow (L/hr)	1200
Collision Gas Flow (mL/min)	ON
Collision Gas	Argon

Marker Names, Retention Times, MRMs, and Tuning Information for Xevo TQ-S

Marker	GAG	Retention Time (RT)	MRM	Cone (V)	Collision
Chondrosine	Internal Standard	1.0 min	355.09 > 194.07	30	14
D0A0	Heparan Sulfate	1.2 min	378.18 > 175.13	30	14
D0S0	Heparan Sulfate	1.3 min	416.11 > 138.08	70	24
D0A6	Heparan Sulfate	1.4 min	457.90 > 174.90	55	22
D0a4	Dermatan Sulfate	1.0 min	457.97 > 300.08	28	22
D0a6	Chondroitin Sulfate	1.2 min	458.00 > 282.00	26	20

STANDARD OPERATING PROCEDURE:
LC-MS/MS MEASUREMENT OF URINARY CREATININE

Calculations for urine dilution were made based on the redwoodtoxicology.com claim that average urinary creatinine levels range from 40-300 mg/dL. This translates to concentrations of 3.5-26.5 mM creatinine in an undiluted urine sample (113.12 g/mol creatinine). A 2500x dilution of undiluted urine samples would yield a possible range of 1.4-10.6 μM creatinine (an acceptable range for measurement via LC-MS).

Dilute liquid urine samples 5x in MilliQ water.

Urine Filter Paper Extraction (*ref: Miki K, Sudo A. 1998. Effect of urine pH, storage time, and temperature on stability of catecholamines, cortisol, and creatinine. Clin. Chem. 44(8): 1759-1762.*):

If urine is provided dried on filter paper, extract the urine using 5x the volume of urine originally saturated in the paper. The amount of volume of water to use may be determined by taking the weight of the urine filter paper and dividing the total volume of urine blotted by the weight of the paper. A fraction of the paper may be cut off, weighed, and the weight of the fraction should be multiplied by the urine volume/paper weight value to determine the original volume of urine saturated in the filter paper. Multiply this number by five to determine the amount of water to use in urine extraction. Store the remaining filter paper under desiccant at -20°C.

$$\frac{Total\ Filter\ Paper\ Weight}{Saturated\ Urine\ Volume} \times Weight\ of\ Filter\ Paper\ Segment \times 5 = Water\ for\ Urine\ Extraction$$

Place the filter paper fragment into a 50 mL falcon tube. If the fragment is too large to be completely submerged in the predetermined volume of water (5x original saturated volume), cut it into smaller pieces. Add the water and mix via vortexing. Allow the samples to sit at 4°C overnight and mix again via vortexing the next morning. Briefly centrifuge the samples (5 seconds at 1,000g) to collect the liquid at the bottom of the tube. Remove the liquid using a 1,000 mL pipette, pressing the paper with the pipette tip to squeeze out as much liquid as possible. Transfer the liquid to a labelled container such as a 15 mL falcon tube or a 1.5 mL microcentrifuge tube for storage at -20°C.

From the 5x diluted liquid urine or 5x diluted urine extract, take 2 μL and bring to a volume of 1.0 mL using MilliQ water to make 2,500x diluted urine samples. Prepare a serially diluted scaffold of standards for linear regression and measurement of creatinine concentration following the procedure below.

Starting with a 20 mM solution of creatinine in water, prepare a set of nine creatinine standards ranging from 250 nM to 15 μM in water. These standards are used to measure the concentration of 2500x diluted urine samples with a formula obtained by linear regression. The concentrations to prepare are 15 μM, 12 μM, 8 μM, 5 μM, 2 μM, 1 μM, 750 nM, 500 nM, and 250 nM.

LC-MS Analysis For Creatinine Measurement in Urine:

Urinary creatinine was measured using a Waters Acquity UPLC with fixed loop injector connected to a Waters Xevo TQ mass spectrometer. Compounds were separated with a Waters Acquity UPLC BEH Shield RP18 1.7 μm 2.1x100 mm column (Waters P/N 186002854) using a 3 minute isocratic method of

50% 0.1% formic acid in water (MPA) and 50% 0.1% formic acid in acetonitrile at a flow rate of 0.2 mL/min. The weak needle wash was 0.1% formic acid in 90/10 water/acetonitrile, the strong needle wash was MPB, and the seal wash was 50/50 water/acetonitrile.

CREATININE MRM/MS TUNE

Analyte	Precursor (m/z)	Product (m/z)	Cone (V)	Collision (V)
Creatinine	114.1	44	40	15
Creatinine-d3	117.1	89	40	15

WATERS XEVO TQ SETTINGS

Parameter	Setting
Polarity	ESI+
Capillary (kV)	3.50
Cone (V)	25.00
Extractor (V)	3.00
Source Temperature (°C)	150
Desolvation Temperature (°C)	400
Cone Gas Flow (L/Hr)	50
Desolvation Gas Flow (L/Hr)	500
Collision Gas Flow (mL/min)	0.15
LM 1 Resolution	3.0
HM 1 Resolution	15.0
LM 2 Resolution	2.8
HM 2 Resolution	15.0
